# More than just visits: Timing, frequency, and determinants of effective antenatal care in Bangladesh - BDHS 2007 to 2017-18

**DOI:** 10.1371/journal.pone.0321686

**Published:** 2025-05-02

**Authors:** Md. Hasibul Islam Jitu, Awan Afiaz, Raaj Kishore Biswas

**Affiliations:** 1 Institute of Statistical Research and Training, University of Dhaka, Dhaka, Bangladesh; 2 Department of Biostatistics, University of Washington, Seattle, Washington, United States of America; 3 School of Health Sciences, Faculty of Medicine and Health, The University of Sydney, New South Wales, Australia; Kandahar University, Faculty of Medicine, AFGHANISTAN

## Abstract

**Background:**

Timely initiation and adequate number of antenatal care (ANC) visits are crucial for ensuring the health and well-being of both pregnant women and their unborn children. Despite recent progress, Bangladesh continues to face challenges in achieving sustainable development goal (SDG-3) related to maternal and neonatal health. This study examines the factors contributing to delayed initiation and a low number of ANC visits, while also evaluating the association between the timing and overall number of ANC visits.

**Data:**

Nationally representative data from the Bangladesh Demographic and Health Surveys (BDHS) conducted in 2007 (n = 3050) and 2017–18 (n = 4544) on women aged 15–49 years.

**Methods:**

We investigated two binary outcome variables: late ANC, defined as the initiation of ANC visits after 12 weeks of gestation, and low ANC, defined as having less than four ANC visits. Geospatial mapping was employed to visualize spatial patterns, followed by survey-weighted logistic regression to identify risk factors associated with late initiation of ANC and low ANC visit frequency. Additionally, classification tree analysis was utilized to explore interactions between predictors and outcomes.

**Results:**

Logistic regression modeling revealed that late ANC was associated with a more than fourfold increase in the odds of having fewer than four ANC visits (AOR: 4.60 [95% CI: 3.69–5.73] in 2007 and AOR: 4.68 [95% CI: 4.00–5.48] in 2017–18). Classification tree analysis further confirmed that late ANC initiation was the most critical predictor of total number of ANC attendance, underscoring the necessity of early ANC initiation to ensure sufficient coverage.

**Conclusion:**

Early initiation of ANC is essential for achieving an adequate number of ANC visits. Notably, the same set of sociodemographic factors remained statistically significant predictors in both 2007 and 2017, highlighting the persistent nature of these disparities and underscoring the urgent need for targeted policies and health interventions.

## Introduction

Timely and adequate access to antenatal care (ANC) is essential for reducing the risks associated with pregnancy and childbirth, thereby improving maternal and neonatal health outcomes [1]. When provided by medical professionals throughout pregnancy, from conception to labor, ANC encompasses comprehensive services, including risk assessment, disease prevention, treatment, and health education [2,3]. In Bangladesh, however, a significant proportion of women delay the initiation of ANC until later stages of pregnancy and fail to attend the recommended number of ANC visits [4]. Therefore, improving the timely initiation and adequacy of ANC is imperative for advancing maternal healthcare in Bangladesh, as it plays a pivotal role in reducing maternal and neonatal risks and improving overall health outcomes.

The timely initiation of ANC in the first trimester is critical for optimizing maternal and fetal health outcomes, as it provides an opportunity for early intervention, risk assessment, and the implementation of preventive measures that support a healthier pregnancy and childbirth [2]. Achieving Sustainable Development Goal 3 requires prioritizing timely and adequate ANC visits, as they are fundamental to reducing maternal mortality (target 3.1) and preventing neonatal and under-5 mortality (target 3.2) [5]. Furthermore, the Global Strategy for Women’s, Children’s, and Adolescents’ Health (2016–2030) seeks to eliminate stillbirths and other preventable deaths among women, children, and adolescents by 2030, emphasizing the critical role of comprehensive and timely healthcare interventions [6]. Timely and sufficient ANC visits can play an important role in achieving this goal.

Prior to 2016, the WHO recommended at least four ANC visits during pregnancy (the FANC model) [7]. The revised 2016 WHO guidelines increased the requirement to at least eight visits [3]. However, Bangladesh’s current national guideline still recommends four visits [4]. The first ANC visit should occur by 12 weeks (WHO 2016 guideline), 8–12 weeks (FANC model), and by 16 weeks (Bangladesh national guideline) [3,4,7].

Previous studies have demonstrated that timely and adequate ANC visits are strongly associated with reduced risks of low birthweight, stillbirth, anemia, perinatal and neonatal mortality, as well as maternal mortality [8–10]. Moreover, routine ANC visits offer valuable opportunities for screening non-communicable diseases, such as diabetes, and provide essential guidance on lifestyle modifications to mitigate risks associated with drug and alcohol misuse, smoking, obesity, and malnutrition [2]. However, home births, often assisted by traditional birth attendants (TBAs), continue to be prevalent in Bangladesh due to economic constraints, traditional beliefs, religious factors, limited access to healthcare facilities, and a lack of awareness about alternative options [11]. These factors may contribute to reduced access to essential ANC visits. Additionally owning mobile phones also significantly plays a critical role in improving maternal health outcomes in low- and middle-income countries (LMICs) like Bangladesh. Studies have found that mobile phones help better access to health information, ANC visits, emergency health communication and improving overall awareness [12,13].

Most existing literature primarily focuses on factors influencing the frequency of ANC visits, while only a limited number of studies, particularly those involving Sub-Saharan African data, have examined the factors that affect the timing of ANC visits [14–18]. This underscores a gap in the literature regarding the relationship between late initiation of ANC and subsequent low ANC attendance. The objective of this study was to address this gap by analyzing the factors associated with delayed and insufficient ANC visits in Bangladesh and examining the impact of late ANC initiation on the overall number of ANC visits, using nationally representative data from 2007 and 2017–18. We specifically wanted to assess the change in ANC service structure in Bangladesh across one decade and thus we chose to use 2007 and 2017–18 survey datasets.

### Theoretical framework

**Fig 1:**
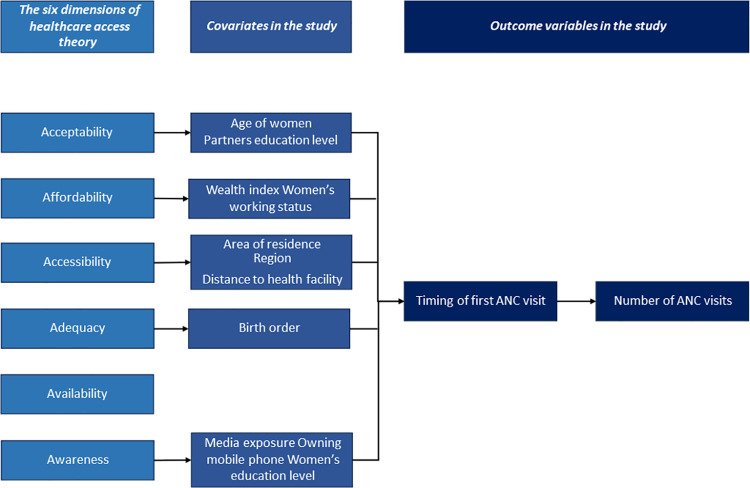
Theorical framework for the current studies regarding timing and number of ANC visit.

This study investigated the factors associated with the low number of ANC visits in Bangladesh through the lens of healthcare access theory. We adopted a modified version of the theoretical framework used by Afiaz & Biswas (2021) [19], who utilized Saurman's (2016) [20] extended version of the original healthcare access theory proposed by Penchansky & Thomas (1981) [21]. Saurman's (2016) [20] extended version included an additional dimension called ‘awareness’ on top of the existing five dimensions of acceptability, availability, affordability, accessibility, and adequacy prescribed by Penchansky & Thomas (1981) [21]. While our analysis explores aspects such as accessibility, acceptability, affordability, adequacy, and awareness of ANC visits, data constraints limit the exploration of the dimension of availability (Fig 1). Our findings aim to inform targeted interventions aimed at optimizing timing and the number of ANC visits, incorporating both hardware and software strategies to address knowledge gaps and promote maternal and child health.

## Methods

### Data overview

The data used in this analysis comes from the Bangladesh Demographic and Health Survey (BDHS). BDHS is a part of the Demographic Health Survey program conducted in LMICs by USAID, collects accurate, nationally representative data on fertility, family planning, maternal and child health, HIV/AIDS, and nutrition [22]. We utilized the Bangladesh Demographic and Health Survey (BDHS) data sets from 2007 (n = 3050) and 2017–18 (n = 4544). Both of these two BDHS 2007 and 2017–18 surveys were chosen to allow for a comparative analysis over a decade, capturing potential changes in antenatal care (ANC) utilization patterns and associated sociodemographic factors over time. The sampling frame for both surveys consisted of a list of enumeration areas (EAs) derived from the census. Employing a two-stage stratified cluster sampling approach with probability proportional to size sampling method, the initial stage comprised selecting 361 EAs in 2007 and 675 EAs in 2017–18. Subsequently, a second stage involved employing systematic sampling method to select an average of 30 households from each EA. In the 2017–18 BDHS, data collection occurred in a total of 20,160 households and 10,819 households made up the sample of data for the 2007 BDHS.

### Outcome variables and covariates

We investigated two binary outcome variables pertaining to the ANC visits. The first outcome variable was for an indicator for whether the timing of the ANC visit was late (after 12 weeks). We will refer to this as late ANC for brevity from here on. The second outcome variable was the indicator for whether the number of ANC visits were ‘less than four’, which will be referred to as low ANC from here on. While Bangladesh national guidelines define late ANC initiation as the first visit occurring after 16 weeks of gestation, this study adopted WHO’s stricter >12-week threshold to align with global standards and enhance cross-LMIC comparability. For ANC frequency (i.e., Low ANC), we analyzed both WHO’s updated 8-visit benchmark and Bangladesh’s 4-visit guideline, prioritizing the latter in primary analyses to reflect local policy and the 2007 and 2017–18 BDHS datasets. Supplemental use of the WHO 8-visit threshold produced directionally consistent results but amplified the effect sizes (detailed in S1, S2, S4, and S5 Tables).

The covariates included in the study were as follows: area of residence, wealth index, partner’s education level, women’s education level, women’s working status, media exposure, distance to health facility, birth order, division/region, women’s age, and owning mobile phone. Note that the divisional structure differed between the 2007 and 2017–18 surveys due to administrative boundary changes in Bangladesh. The Dhaka division was divided into Mymensingh and Dhaka, while the Rajshahi division was divided into Rangpur and Rajshahi. Detailed information on this is provided in the supplementary document (S1 Text).

### Statistical analysis

Given that the study utilized large-scale survey data, missing cases were handled using listwise deletion, assuming that the data were missing at random to ensure unbiased estimates [23,24]. An initial summary of the data was provided by a descriptive statistics table, accompanied by a visualization of the spatial distribution of late ANC initiation and low ANC attendance through descriptive statistics and geographical mapping. To examine the factors associated with these outcomes, complex survey-weighted adjusted logistic regression models with a quasi-binomial family distribution were employed, enabling the analysis of binary outcomes while accounting for overdispersion. Multicollinearity was assessed using generalized variance inflation factor (GVIF) scores for each predictor.

We utilized classification tree analysis to investigate complex interactions between predictors and nonlinear relationships between predictor variables and the outcome measures [25]. It partitions data using hypothesis testing at each node. The null hypothesis is $H_{0j}:D\left(\text{\textbackslash{}textit\{Y}\}|X_{j}\right)=D\left(\text{\textbackslash{}textit\{Y}\}\right)$, which assesses whether the response variable \textit{Y} is independent of each covariate $X_{j};$ j = 1, 2, …, m. The test statistics is defined as:

$T_{j}\left(L_{n},w\right)=\text{vec}\left(\sum_{i=1}^{n}w_{i}g_{j}\left(X_{ji}\right)h\left(Y_{i},{(Y}_{1},Y_{2},\ldots ,Y_{n}\right){)}^{\prime }\right),$

where $g_{j}\left(X_{ji}\right)$ is a transformation of the predictor, $h\left(Y_{i},{(Y}_{1},Y_{2},\ldots ,Y_{n}\right){)}^{\prime }$ represents the influence function of the response, and $w_{i}$ denotes case weight. The test statistic is standardized using its conditional expectation ($\mu_{j}$) and covariance ($\Sigma_{j}$) under the null hypothesis. If $H_{0j}$ is rejected, the algorithm selects the covariate with the strongest association (smallest p-value). In the next step it determines the optimal split by maximizing a discrepancy measure:


$$A^{*}=arg\;\max_{A}\;c\left(T_{A},\mu_{A},\Sigma_{A}\right)$$

The recursion halts when no significant association is found at a predefined significance level (α).

The results of the classification tree analysis provide useful insights. The tree structure visually interprets the importance of covariates in splitting data. The significance of splits is determined by p-values, with corrections like Bonferroni adjustments applied to handle multiple comparisons. Terminal nodes provide a summary of the response variable within the distinct partitions.

Statistical software R (version 4.3.1) [26] was used for performing all statistical analyses, including creating the geographical maps in this study. The analysis utilized several R packages: tidyverse [27], survey [28], ggplot2 [29], partykit [25] and gridExtra [30]. Besides, from an openly licensed database called geoBoundaries Global Administrative Database, we obtained the administrative boundary shapefile of Bangladesh [31].

### Ethical Considerations

This work does not include any studies with human participants conducted by the authors. Therefore, no ethical approval was needed for our study as we are using secondary data sets provided by the Demographic Health Survey (DHS). The survey protocol for the Bangladesh DHS (BDHS) datasets utilized in this study received approval by institutional review boards (IRBs) at ICF and the Bangladesh Medical Research Council (BMRC). Written informed consent was obtained from each participant before conducting the interviews. Participants who did not provide consent were excluded from the survey. Participants were made aware that their participation was entirely voluntary. They were assured that their responses would be kept confidential and anonymous. Additionally, they were informed of their right to skip any questions they preferred not to answer and could withdraw from the interview at any point. All data were fully anonymized before the authors were given access. The datasets are available on request on the Demographic and Health Survey (DHS) website free of cost for research purposes (https://dhsprogram.com/).

## Results

### Descriptive analysis

The distribution of late initiation of ANC and less than four ANC visits by sociodemographic factors is shown in Table 1. In both 2007 and 2017–18, approximately 60% of women had a delayed first ANC visit. Meanwhile, low ANC was 65.8% in 2007 and decreased to 48.8% in 2017, which shows that over the ten years, late ANC remained same, but low ANC decreased by 17%. In 2007 and 2017–18, respectively, 80.2% and 64.8% of women who started their ANC visit late also had less than four ANC visits, whereas women who were not late, respectively, 55.4% and 74.9%, had over four ANC visits. In both 2007 and 2017–18, rural women had higher late ANC visits (15.2% and 13.3%, respectively) and also reported higher low ANC visits (22.2% and 15.2%, respectively) than women in urban areas. The poorest, less educated, and non-media-exposed women had more late ANC and low ANC visits compared to the other categories despite the survey years. In 2017–18 data, women who reported that the distance to health facilities was a problem had, respectively, 7.4% and 10.6% higher late ANC and low ANC visits compared to the women who had no issue with the distance to the health facility. Women whose most recent birth was of order four or higher reported greater percentage of late and low ANC visits compared to the women giving birth to a first, second, or third child. On the other hand, women who owned mobile phones reported 13.1% and 14.3% lower percentage of late and low ANC visits compared with women who did not own one. Women with partners with higher education also reported a lower percentage of late and low ANC visitscompared to those with primary or no education.

**Table 1:** The frequency distribution of late ANC and low ANC (< 4) visits by sociodemographic factors*.*

Variables	BDHS 2007			BDHS 2017–18		
	Total sample, N = 2,949 (%)	N (%) of women with Late ANC visits	N (%) of women with Low (< 4) ANC visits	Total sample, N = 4,588 (%)	N (%) of women with Late ANC visits	N (%) of women with Low (< 4) ANC visits
**Area of residence**						
Urban	783 (26.5)	379 (48.4)	387 (49.5)	1,266 (27.6)	634 (50.0)	479 (37.8)
Rural	2,166 (73.5)	1,377 (63.6)	1,553 (71.7)	3,322 (72.4)	2,104 (63.3)	1,761 (53.0)
**Wealth index**						
Poorest	446 (15.1)	325 (72.9)	357 (80.2)	848 (18.5)	620 (73.2)	531 (62.6)
Poorer	495 (16.8)	339 (68.5)	393 (79.4)	912 (19.9)	605 (66.3)	539 (59.0)
Middle	549 (18.6)	354 (64.6)	410 (74.7)	894 (19.5)	561 (62.8)	459 (51.4)
Richer	688 (23.3)	397 (57.8)	433 (62.9)	970 (21.2)	582 (60.0)	447 (46.0)
Richest	772 (26.2)	340 (44.1)	347 (45.0)	964 (21.0)	369 (38.2)	265 (27.4)
**Region**						
Dhaka	882 (29.9)	505 (57.3)	575 (65.3)	1,185 (25.8)	595 (50.2)	531 (44.8)
Barishal	164 (5.6)	95 (57.6)	113 (68.6)	241 (5.3)	155 (64.2)	135 (55.8)
Chattogram	617 (20.9)	343 (55.5)	416 (67.4)	966 (21.1)	633 (65.5)	556 (57.5)
Khulna	356 (12.1)	214 (60.1)	230 (64.7)	439 (9.6)	263 (60.0)	177 (40.3)
Mymensingh				381 (8.3)	228 (59.8)	189 (49.6)
Rajshahi	721 (24.4)	479 (66.4)	446 (61.8)	550 (12.0)	366 (66.4)	273 (49.5)
Rangpur				502 (10.9)	322 (64.0)	187 (37.2)
Sylhet	209 (7.1)	121 (58.1)	161 (77.0)	323 (7.0)	176 (54.4)	192 (59.4)
**Women’s age (Mean [SD])**	24.9 (5.8)	24.5 (5.8)	24.8 (5.9)	24.8 (5.5)	24.7 (5.6)	24.8 (5.6)
**Women’s education level**						
No education	474 (16.1)	311 (65.7)	389 (82.0)	229 (5.0)	166 (72.7)	166 (72.7)
Primary	828 (28.1)	541 (65.3)	636 (76.8)	1,198 (26.1)	841 (70.2)	731 (61.0)
Secondary	1,340 (45.4)	787 (58.8)	816 (60.9)	2,314 (50.4)	1,404 (60.7)	1,087 (47.0)
Higher	307 (10.4)	117 (38.0)	100 (32.4)	847 (18.5)	326 (38.5)	256 (30.2)
**Women’s employment status**						
Not Working	2,193 (74.4)	1,293 (58.9)	1,423 (64.9)	2,919 (63.6)	1,653 (56.6)	1,419 (48.6)
Working	756 (25.6)	464 (61.3)	517 (68.4)	1,669 (36.4)	1,084 (64.9)	821 (49.2)
**Partner’s education level**						
No education	734 (24.9)	516 (70.3)	588 (80.1)	576 (12.5)	421 (73.2)	369 (64.0)
Primary	775 (26.3)	483 (62.3)	570 (73.6)	1,488 (32.4)	1,025 (68.9)	864 (58.1)
Secondary	964 (32.7)	562 (58.3)	596 (61.8)	1,617 (35.3)	944 (58.4)	761 (47.0)
Higher	476 (16.1)	195 (41.0)	187 (39.2)	907 (19.8)	347 (38.2)	247 (27.2)
**Media exposure**						
No	795 (27.0)	532 (66.9)	619 (77.8)	1,458 (31.8)	1,021 (70.0)	909 (62.4)
Yes	2,154 (73.0)	1,225 (56.9)	1,322 (61.4)	3,130 (68.2)	1,717 (54.8)	1,331 (42.5)
**Birth order**						
1	1,162 (39.4)	669 (57.6)	701 (60.4)	1,815 (39.6)	1,007 (55.5)	795 (43.8)
2-3	1,298 (44.0)	795 (61.3)	849 (65.4)	2,276 (49.6)	1,373 (60.3)	1,109 (48.7)
4+	489 (16.6)	292 (59.7)	390 (79.8)	498 (10.8)	358 (71.9)	336 (67.5)
**Distance to health facility**						
Not a big problem				2,758 (60.1)	1,563 (56.7)	1,230 (44.6)
Big problem				1,830 (39.9)	1,174 (64.14)	1,010 (55.2)
**Owning mobile phone**						
No				1,670 (36.4)	1,136 (68.0)	968 (57.9)
Yes				2,918 (63.6)	1,602 (54.9)	1,272 (43.6)
**Timing of first ANC visit**						
Not late	1,193 (40.4)		532 (44.6)	1,851 (40.3)		465 (25.1)
Late	1,756 (59.6)		1,409 (80.2)	2,737 (59.7)		1,775 (64.8)
**Number of ANC visit**						
≥4 ANC	1009 (34.2)			2,348 (51.2)		
Low (<4) ANC	1,940 (65.8)			2,240 (48.8)		

The distribution of late initiation of ANC and less than eight ANC visits by sociodemographic factors, as well as the frequency distribution of low ANC visits (<8) by late ANC is provided supplementary document (S1 and S2 Tables).

### Logistic regression models

The results of binary logistic regression model adjusted for sociodemographic factors with timing of first ANC visit and number of ANC visits (low (< 4) ANC visits) as outcome is shown in Table 2. Among urban and rural mothers, in 2007, rural mothers were associated with 26% higher odds (AOR: 1.26 [95% CI: 1.03, 1.53]; p-value = 0.023) of having a late ANC visit compared to urban mothers. In terms of the number of ANC visits, rural mothers were associated with 30% (AOR: 1.30 [1.08, 1.55]; p-value = 0.005) and 71% (AOR: 1.71 [1.38, 2.11]; p-value < 0.001) higher odds of having low ANC visits, respectively, in 2017–18 and 2007, compared to urban mothers. For each one-year difference in women’s age, older women were associated with 2% lower (AOR: 0.98 [95% CI: 0.96, 1.00]; p-value = 0.036) and 3% lower odds (AOR: 0.97 [95% CI: 0.95, 0.99]; p-value = 0.003) of having a late ANC visit in 2017–18 and 2007, respectively. Similarly, the odds of having a low number of ANC visits were found lower by 3% for each one year of age in both 2017–18 (AOR: 0.97 [95% CI: 0.95, 0.99]; p-value < 0.001) and 2007 (AOR: 0.97 [95% CI: 0.95, 0.99]; p-value = 0.011). Despite the survey year, the poorest, least educated, and, in 2017–18 non-media-exposed women were associated with higher odds of having late and low number of ANC visits compared to the reference categories. Besides, for 2017–18, those for whom distance to health facilities was a problem were associated with 20% higher odds (AOR: 1.20 [1.03, 1.38]; p-value = 0.016) of having low ANC visits compared to those who did not have any problem with the distance. In the case of women owning mobile phones, there were associated with, respectively, 16% (AOR: 0.84 [0.72, 0.97]; p-value = 0.021) and 23% (AOR: 0.77 [0.66, 0.89]; p-value < 0.001) lower odds of having late and low ANC visits compared to women without a mobile phone. Irrespective of the survey year, in the case of the highest birth order, mothers were associated with higher odds of having late and low ANC visits compared to if they were giving birth to a first, second, or third child. Women with higher-educated partners were also associated with lower odds of having late and low ANC coverage compared to those having partners with primary or no education.

**Table 2:** Binary logistic regression model adjusted for sociodemographic factors with timing of first ANC visit and number of ANC visits (low (< 4) ANC visits) as outcome*.*

Variables	Late ANC visits						Low (< 4) ANC visits					
	BDHS 2007			BDHS 2017–18			BDHS 2007			BDHS 2017–18		
	AOR	95% CI	p-value	AOR	95% CI	p-value	AOR	95% CI	p-value	AOR	95% CI	p-value
**Area of residence**												
Urban (ref.)	—	—		—	—		—	—		—	—	
Rural	1.26	1.03, 1.53	**0.023**	1.14	0.97, 1.35	0.122	1.71	1.38, 2.11	**<0.001**	1.30	1.08, 1.55	**0.005**
**Wealth index**												
Poorest (ref.)	—	—		—	—		—	—		—	—	
Poorer	0.85	0.60, 1.20	0.358	0.88	0.69, 1.12	0.296	1.04	0.69, 1.56	0.842	1.06	0.83, 1.34	0.658
Middle	0.78	0.56, 1.09	0.140	0.91	0.71, 1.18	0.489	0.93	0.63, 1.39	0.739	0.86	0.68, 1.10	0.236
Richer	0.66	0.47, 0.92	**0.014**	1.00	0.76, 1.31	0.981	0.68	0.46, 1.01	0.058	0.79	0.61, 1.02	0.074
Richest	0.49	0.34, 0.72	**<0.001**	0.58	0.43, 0.79	**<0.001**	0.49	0.31, 0.76	**0.002**	0.46	0.34, 0.63	**<0.001**
**Region**												
Dhaka (ref.)	—	—		—	—		—	—		—	—	
Barishal	0.83	0.62, 1.13	0.238	1.40	1.07, 1.82	**0.013**	0.87	0.62, 1.23	0.422	1.05	0.80, 1.36	0.742
Chattogram	0.93	0.73, 1.19	0.556	1.73	1.38, 2.17	**<0.001**	1.09	0.84, 1.43	0.516	1.43	1.14, 1.81	**0.002**
Khulna	1.03	0.77, 1.37	0.845	1.29	0.99, 1.68	0.063	0.88	0.65, 1.19	0.410	0.66	0.50, 0.88	**0.004**
Mymensingh				1.09	0.82, 1.44	0.563				0.77	0.58, 1.02	0.069
Rajshahi	1.25	0.96, 1.63	0.093	1.63	1.24, 2.14	**<0.001**	0.65	0.48, 0.87	**0.004**	0.92	0.71, 1.20	0.542
Rangpur				1.32	0.99, 1.74	0.055				0.44	0.33, 0.58	**<0.001**
Sylhet	0.89	0.63, 1.25	0.508	0.79	0.61, 1.02	0.071	1.28	0.88, 1.88	0.199	1.09	0.84, 1.41	0.510
**Women’s age**	0.97	0.95, 0.99	**0.003**	0.98	0.96, 1.00	**0.036**	0.97	0.95, 0.99	**0.011**	0.97	0.95, 0.99	**<0.001**
**Women’s education level**												
No education (ref.)	—	—		—	—		—	—		—	—	
Primary	1.04	0.78, 1.40	0.787	0.93	0.65, 1.34	0.702	0.89	0.62, 1.28	0.517	0.62	0.42, 0.89	**0.010**
Secondary	0.98	0.71, 1.36	0.911	0.76	0.53, 1.09	0.130	0.57	0.39, 0.83	**0.004**	0.48	0.33, 0.70	**<0.001**
Higher	0.77	0.49, 1.20	0.250	0.52	0.35, 0.78	**0.002**	0.36	0.22, 0.59	**<0.001**	0.45	0.29, 0.70	**<0.001**
**Women’s employment status**												
Not Working (ref.)	—	—		—	—		—	—		—	—	
Working	0.93	0.76, 1.15	0.524	1.08	0.94, 1.26	0.281	1.03	0.83, 1.29	0.768	0.85	0.73, 0.99	**0.039**
**Partner’s education level**												
No education (ref.)	—	—		—	—		—	—		—	—	
Primary	0.75	0.58, 0.97	**0.030**	0.92	0.71, 1.20	0.558	0.88	0.65, 1.18	0.385	0.99	0.78, 1.25	0.944
Secondary	0.79	0.60, 1.04	0.098	0.73	0.56, 0.96	**0.023**	0.76	0.56, 1.03	0.078	0.82	0.64, 1.05	0.120
Higher	0.52	0.36, 0.76	**<0.001**	0.49	0.36, 0.68	**<0.001**	0.53	0.37, 0.77	**<0.001**	0.53	0.39, 0.73	**<0.001**
**Media exposure**												
No (ref.)	—	—		—	—		—	—		—	—	
Yes	0.98	0.77, 1.23	0.848	0.79	0.67, 0.94	**0.008**	0.98	0.75, 1.28	0.885	0.72	0.60, 0.85	**<0.001**
**Birth order**												
1 (ref.)	—	—		—	—		—	—		—	—	
2-3	1.29	1.04, 1.60	**0.022**	1.17	0.97, 1.42	0.097	1.25	0.99, 1.57	0.061	1.32	1.10, 1.59	**0.003**
4+	1.19	0.82, 1.72	0.357	1.55	1.08, 2.24	**0.018**	1.92	1.26, 2.93	**0.002**	2.31	1.64, 3.24	**<0.001**
**Distance to health facility**												
Not a big problem (ref.)				—	—					—	—	
Big problem				1.1	0.94, 1.27	**0.233**				1.20	1.03, 1.38	**0.016**
**Owning mobile phone**												
No (ref.)				—	—					—	—	
Yes				0.84	0.72, 0.97	**0.021**				0.77	0.66, 0.89	**<0.001**

AOR = Adjusted Odds Ratio, CI = Confidence Interval

In 2017–18, women who reported late ANC initiation were associated with 4.68 times (AOR: 4.68 [95% CI: 4.00, 5.48]; p-value < 0.001) the odds of having less than four ANC visits compared to those who were not late after adjusting for the others covariate, which remained unchanged in 2007 (AOR: 4.60 [95% CI: 3.69, 5.73]; p-value < 0.001), shown in Table 3 and detailed in S3 Table.

**Table 3:** Binary logistic regression model adjusted for sociodemographic factors and timing of first ANC visit with number of ANC visits (low (< 4) ANC visits) as outcome.

Characteristic	BDHS 2007			BDHS 2017–18		
	AOR	95% CI	p-value	AOR	95% CI	p-value
**Timing of first ANC visit**						
Not late (ref.)	—	—		—	—	
Late	4.60	3.69, 5.73	**<0.001**	4.68	4.00, 5.48	**<0.001**

AOR, adjusted odds ratio; CI, confidence interval.

Adjusted for area of residence, wealth index, region, women’s age, women’s education level, women’s employment status, partner’s education level, media exposure, birth order, distance to health facility, and owning mobile phone.

The supplementary document (S4 and S5 Tables) provides binary logistic regression models adjusted for sociodemographic factors and the timing of the first ANC visit, with number of ANC visits as outcome categorized as fewer than eight ANC visits or more than eight ANC visits.

### Spatial mapping

**Fig 2:**
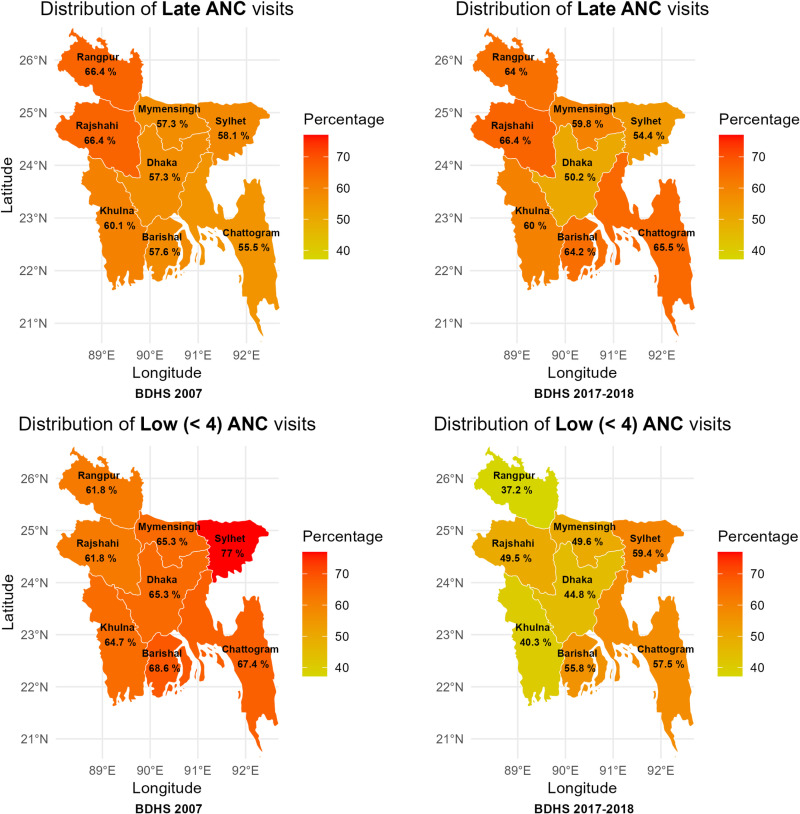
Region-wise percentage distribution of late and low (< 4) ANC visit in Bangladesh.

The map presented in Fig 2 illustrates the regional distribution of late and low (< 4) ANC visits in Bangladesh. In 2007, Rajshahi and Rangpur regions showed the highest percentage of late ANC visits at 66.4%, while Chattogram region had the lowest at 55.5%. Conversely, in 2017–18, Rajshahi region maintained the highest percentage of late ANC visits at 66.4%, with Dhaka region recording the lowest at 50.2%. This indicates notable changes in the regional distribution of late ANC visits over the decade, with nearly half of the regions showing improvement while others worsening.

Regarding low ANC visits, in 2007, the Sylhet division had the highest percentage at 77%, while Rajshahi and Rangpur regions had the lowest at 61.8%. By 2017–18, Sylhet region still had the highest percentage at 59.4%, whereas Rangpur region had the lowest at 37.2%. This suggests improvement across almost all regions in terms of the number of ANC visits over the decade.

### Classification trees

**Fig 3:**
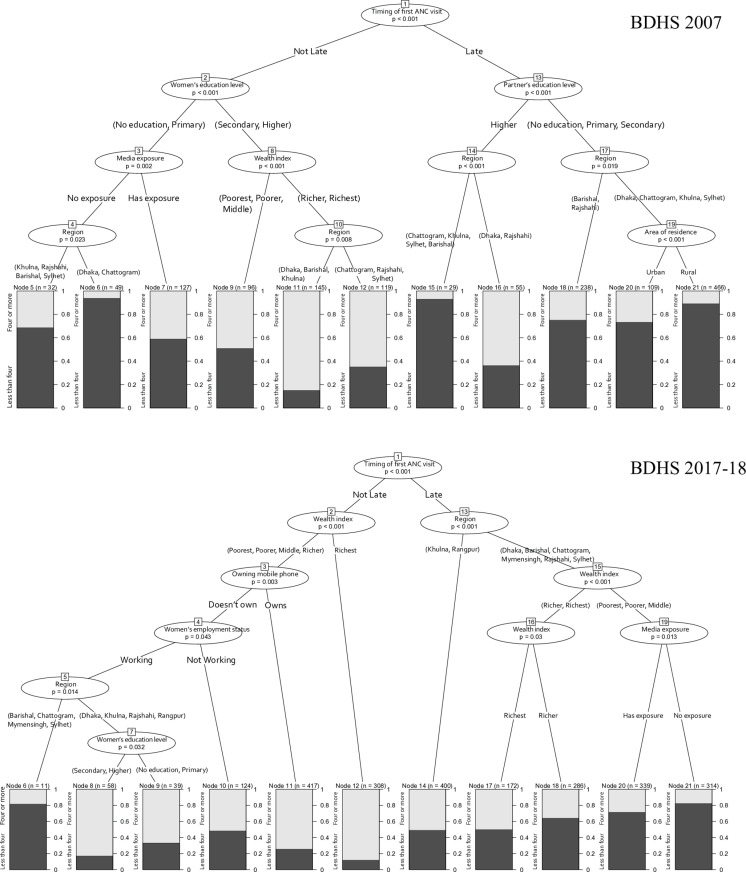
Classification tree illustrating the important predictors of low ANC visits in Bangladesh for 2007 and 2017-18.

According to the classification trees (Fig 3), in both 2007 and 2017–18, the most important predictor of the low number of ANC visits was the timing of the first ANC visit appearing at the top of the tree. Administrative divisions (region) also remained very important across the two time points. Notice that most of the nodes on the right (which corresponded to late ANC initiation) had a higher prevalence of low ANC (< 4), which highlighted the importance of early initiation in ensuring adequate ANC coverage. Wealth index, mobile ownership, media exposure also became prominent for the most recent survey data and coincided with our earlier findings from the regression models.

### Model validation

The supplementary document (S6, S7, S8, S9, and S10 Tables) includes generalized variance inflation factors (GVIF) analysis for binary logistic regression models to assess multicollinearity. All the squared adjusted GVIF values are below five, indicating that our regression models do not have multicollinearity issues [32].

## Discussion

Our study explored the association between late initiation of ANC and subsequent low ANC attendance, highlighting the critical importance of early ANC visits and their relationship with achieving the recommended number of ANC visits. We identified sociodemographic factors associated with both timing and total number of ANC visits. The findings revealed that, in both 2007 and 2017–18, approximately 60% of women delayed their first ANC visit, with a slight increase observed over the decade. While crude prevalence estimates for low ANC visits decreased by 17%, nearly half of the women (approximately 50%) still did not meet the minimum number of ANC visits recommended by the WHO FANC model and national guidelines in Bangladesh. Importantly, the factors associated with both late and insufficient ANC visits remained largely unchanged over the 10-year period. Our results underscore that, despite governmental efforts to improve maternal healthcare, sociodemographic factors such as education, socioeconomic status, maternal age, and birth order have continued to influence the timing and frequency of ANC visits throughout this period. Furthermore, Bangladesh national guidelines need to revisit the recommended number and timing of ANC visits to align with WHO standards. This would enhance the understanding, help in cross-country comparisons and adapting global strategies to improve maternal health outcomes.

Our analysis revealed a strong association between the timing of the first ANC visit and the subsequent number of ANC visits, as evidenced by the regression model results. The classification tree analysis further confirmed that the timing of the first ANC visit was the most significant predictor of number of ANC visits, with consistent findings across both the 2007 and 2017–18 surveys. These results are particularly pertinent to LMICs such as Bangladesh, where traditional societal norms and cultural beliefs exert a substantial influence on healthcare-seeking behaviors [33,34]. In Bangladeshi rural areas, pregnancy is often regarded as a natural process that does not require early medical intervention unless complications arise, leading to delayed ANC initiation [35]. Additionally, decision-making regarding maternal health is frequently influenced by husbands or elder family members [33,35], who may not prioritize early ANC visits due to traditional views or a lack of awareness. Furthermore, rural women, who are the most susceptible to being influenced by traditional norms, also showed higher odds of late and low ANC visits compared to urban mothers, irrespective of the survey year, a trend supported by studies in Bangladesh [36] and Vietnam [37]. Additionally, women facing challenges with distance to healthcare facilities showed higher odds of having a low number of ANC visits. Studies conducted in Burkina Faso [38], and Benin [39] further support to this finding.

Despite increases in women’s education and employment, many women in Bangladesh continue to face significant barriers in accessing healthcare and ANC. Issues including cultural norms, poor healthcare facilities, and financial challenges make it difficult for women in accessing healthcare facilities [40]. Women from less affluent households had higher odds of having late initiation and a low number of ANC visits compared to the others, indicating socioeconomic inequalities in ANC coverage in Bangladesh. Financial barriers significantly impact access to maternal healthcare services, as lower-income families may prioritize immediate basic needs over healthcare expenses, leading to delayed or irregular ANC visits [41,42]. Evidence from studies conducted in Ethiopia [43,44], and Cameroon [17] also supports this conclusion. While most regions have shown improvement over the decade, regional disparities still exist, highlighting the ongoing need to address regional inequalities in maternal healthcare access in Bangladesh, as evident from recent studies [45]. Regional variations in ANC utilization also evident in other LMICs like Ethiopia [46] and Indonesia [47]. Women with higher education levels had reduced odds of experiencing late initiation and insufficient ANC visits compared to those with lower levels of education which underscored the crucial role of mother’s education in promoting timely and adequate ANC attendance [41,48,49]. This assertion finds additional backing from research conducted in other LMICs including Nigeria [50,51], Ethiopia [15,46], and Indonesia [47].

Our findings underscored the importance of accessing information and healthcare facilities in ensuring timely and adequate ANC visits. Access to information through media and mobile phones enhances awareness and understanding of the importance of early and regular ANC visits, ultimately improving maternal healthcare utilization [41,48], highlighting the importance of media exposure, internet use, and mobile phones on reproductive healthcare services. A recent study in Bangladesh also highlights the crucial role of internet use, and mobile phone on reproductive health service [52]. Maternal age also has been identified as a significant factor associated with both the timing and number of ANC visits. Previous study conducted in Ethiopia [53], Ghana [54], and Gambia [55] also found maternal age associated with timing and number of ANC visit.

We found that women who gave birth to three or more children previously had increased odds of experiencing late initiation and <4 ANC visits compared to those who had less than two children. This phenomenon can be attributed to several factors. Firstly, mothers with multiple children often face increased household responsibilities [56] and time constraints, which can limit their ability to prioritize and attend regular ANC visits. Additionally, with subsequent births, mothers may consider themselves more experienced and might underestimate the importance of early and frequent ANC visits [57]. This self-satisfaction can lead to reduced utilization of maternal health services [58]. This assertion finds additional backing from research conducted in Ethiopia [44], and Cameroon [17]. Although the study is cross-sectional, valuable policy insights can be drawn from the findings. Strengthening community-based interventions, such as targeted health education campaigns and home visits by community health workers, can improve awareness and encourage early ANC initiation. Integrating ANC services within existing maternal and child health programs and ensuring financial and geographic accessibility, particularly for socioeconomically disadvantaged groups, may reduce barriers to care. Recognizing women’s right to access healthcare during pregnancy and childbirth is essential to reducing the risk of complications and ensuring equitable maternal health outcomes. Policymakers should consider revising national ANC guidelines to emphasize early initiation and continuity of care at the global standard, ensuring they are effectively disseminated and implemented across all healthcare facilities.

The primary strength of our study was that we analyzed nationally representative data spanning over a decade, from 2007 to 2017–18. This extensive timeframe enabled us to explore the persistent factors contributing to late initiation and insufficient ANC visits among women in Bangladesh, which remain unresolved and urgently require attention. Notably, this is the first study of its kind to explore this relationship, utilizing multiple large, nationally representative datasets from Bangladesh Demographic and Health Survey (BDHS) pertaining to Bangladesh. Therefore, it paves the way for further studies in other LMICs in the future. However, several limitations should be noted. First, our study is observational and based on repeated surveys of different, yet representative, cohorts, which limits causal interpretation. Additionally, relying on self-reported data may introduce recall biases or social desirability biases. Some participants may not accurately recall the date of first ANC visit or may over or underreport the number of ANC visits. This recall bias regarding the timing and frequency of ANC visits in self-reported data could affect the validity of the findings. Future studies with health linkage or health insurance data could validate self-reported data with medical records or conducting longitudinal data collection to prevent recall bias. Employing a longitudinal design could better assess causal relationships and evaluate potential intervention strategies based on our findings. Finally, future research can consider applying models such as Spatial Lag Models to address spatial autocorrelation of ANC services in Bangladesh.

## Conclusion

Our study provides valuable insights into ANC utilization among women in Bangladesh from 2007 to 2017–18. We found that the timing of the first ANC visit was strongly associated with whether women received ≥ 4 ANC visits throughout their pregnancy, underscoring the critical importance of early engagement with healthcare services. Despite ongoing efforts to promote maternal health, persistent demographic, socioeconomic, and regional disparities in ANC coverage remain. Women from rural areas, in particular, face barriers such as limited education, financial constraints, and cultural influences, which hinder their access to timely and ≥ 4 ANC visits compared to their urban counterparts. Addressing these disparities requires targeted policies that focus on enhancing maternal education, improving healthcare infrastructure, and promoting community-based initiatives that challenge traditional norms and raise awareness about the benefits of timely ANC visits.

## Supporting information

S1 Table The frequency distribution of late ANC and low (< 8) ANC visits by sociodemographic factors. (DOCX)

S2 Table The frequency distribution of low (<8) ANC visits by timing of first ANC visits. (DOCX)

S3 Table Binary logistic regression model adjusted for sociodemographic factors and timing of first ANC visit with number of ANC visits (low (<4) ANC visits) as outcome. (DOCX)

S4 Table Binary logistic regression model adjusted for sociodemographic factors with timing of first ANC visit and number of ANC visits (low (<8) ANC visits) as outcome. (DOCX)

S5 Table Binary logistic regression model adjusted for sociodemographic factors and timing of first ANC visit with number of ANC visits (low (<8) ANC visits) as outcome. (DOCX)

S6 Table GVIF for binary logistic regression model adjusted for sociodemographic factors with timing of first ANC visit as outcome. (DOCX)

S7 Table GVIF for binary logistic regression model adjusted for sociodemographic factors with number of ANC visits (low (<4) ANC visits) as outcome. (DOCX)

S8 Table GVIF for binary logistic regression model adjusted for sociodemographic factors and timing of first ANC visit with number of ANC visits (low (<4) ANC visits) as outcome. (DOCX)

S9 Table GVIF for binary logistic regression model adjusted for sociodemographic factors number of ANC visits (low (<8) ANC visits) as outcome. (DOCX)

S10 Table GVIF for binary logistic regression model adjusted for sociodemographic factors and timing of first ANC visit with number of ANC visits (low (<8) ANC visits) as outcome. (DOCX)

S1 Text Outcome variables and covariates. (DOCX)

## References

[1] Amponsah-Tabi S, Dassah ET, Asubonteng GO, Ankobea F, Annan JJK, Senu E, et al. An assessment of the quality of antenatal care and pregnancy outcomes in a tertiary hospital in Ghana. PLoS One. 2022;17(10):e0275933. doi: 10.1371/journal.pone.0275933 36223426 PMC9555636

[2] Moller A, Petzold M, Chou D, Say L. Early antenatal care visit: a systematic analysis of regional and global levels and trends of coverage from 1990 to 2013. Lancet Global Health. 2017;5(10). 10.1016/S2214-109X(17)30325-X PMC5603717 28911763

[3] Tunçalp Ӧ., Pena-Rosas JP, Lawrie T, Bucagu M, Oladapo OT, Portela A, et al. WHO recommendations on antenatal care for a positive pregnancy experience-going beyond survival. BJOG. 2017;124(6):860–2. doi: 10.1111/1471-0528.14599 28190290

[4] Sarker BK, Rahman M, Rahman T, Rahman T, Khalil JJ, Hasan M, et al. Status of the WHO recommended timing and frequency of antenatal care visits in Northern Bangladesh. PLoS One. 2020;15(11):e0241185. doi: 10.1371/journal.pone.0241185 33151964 PMC7644040

[5] United Nations. Transforming Our World: the 2030 Agenda for Sustainable Development United Nations United Nations Transforming Our World: the 2030 Agenda for Sustainable Development. United Nations. 2015

[6] Kuruvilla S, Bustreo F, Kuo T, Mishra CK, Taylor K, Fogstad H, et al. The Global strategy for women’s, children’s and adolescents’ health (2016-2030): a roadmap based on evidence and country experience. Bull World Health Organ. 2016;94(5):398–400. doi: 10.2471/BLT.16.170431 27147772 PMC4850541

[7] WHO. WHO Antenatal Care Randomized Trial: Manual for the Implementation of the New Model. Geneva: World Health Organization. 2002.

[8] Hoque AM, Buckus S, Hoque M, Van Hal G. Deciphering the Association of Antenatal Care Visits and Pregnancy Outcomes from South Africa. EJMED. 2022;4(3):175–81. doi: 10.24018/ejmed.2022.4.3.1113

[9] Tibambuya BA, Ganle JK, Ibrahim M. Anaemia at antenatal care initiation and associated factors among pregnant women in West Gonja District, Ghana: a cross-sectional study. Pan Afr Med J. 2019;33:325. doi: 10.11604/pamj.2019.33.325.17924 31692871 PMC6815505

[10] Lima Figueiredo ER, do Socorro Carvalho Miranda C, Viana Campos AC, de Campos Gomes F, Câmara Rodrigues CN, de Melo-Neto JS. Influence of sociodemographic and obstetric factors on maternal mortality in Brazil from 2011 to 2021. BMC Womens Health. 2024 Dec 1;24(1). 10.1186/s12905-024-02925-3 PMC10835861 38302949

[11] Sarker BK, Rahman M, Rahman T, Hossain J, Reichenbach L, Mitra DK. Reasons for Preference of Home Delivery with Traditional Birth Attendants (TBAs) in Rural Bangladesh: A Qualitative Exploration. PLoS One. 2016;11(1):e0146161. doi: 10.1371/journal.pone.0146161 26731276 PMC4701391

[12] Mistry SK, Akter F, Yadav UN, Hossain MB, Sichel A, Labrique AB, et al. Factors associated with mobile phone usage to access maternal and child healthcare among women of urban slums in Dhaka, Bangladesh: a cross-sectional study. BMJ Open. 2021;11(4):e043933. doi: 10.1136/bmjopen-2020-043933 33837099 PMC8043001

[13] Kibria GMA, Hashan MR, Hanif AAM, Maniar V, Shawon MSR. Mobile phone use for pregnancy-related healthcare utilization and its association with optimum antenatal care and hospital delivery in Bangladesh. PLOS Glob Public Health. 2023;3(4):e0001762. doi: 10.1371/journal.pgph.0001762 37022996 PMC10079009

[14] Abera E, Azanaw J, Tadesse T, Endalew M. Prevalence and associated factors of delay antenatal care at public health institutions in Gondar city, Northwest Ethiopia, 2021: a cross-sectional study. Contracept Reprod Med. 2023;8(1):2. doi: 10.1186/s40834-022-00197-6 36647075 PMC9841718

[15] Tesfu A, Aweke A, Gela G, Wudineh K, Beyene F. Factors associated with timely initiation of antenatal care among pregnant women in Bahir Dar city, Northwest Ethiopia: Cross-sectional study. Nursing Open. 2022;9(2). 10.1002/nop2.1162 PMC8859086 34951142

[16] Tumwizere G, K Mbonye M, Ndugga P. Determinants of late antenatal care attendance among high parity women in Uganda: analysis of the 2016 Uganda demographic and health survey. BMC Pregnancy Childbirth. 2024;24(1):32. doi: 10.1186/s12884-023-06214-z 38183021 PMC10768297

[17] Ahinkorah B, Seidu A, Budu E, Mohammed A, Adu C, Agbaglo E, et al. Factors associated with the number and timing of antenatal care visits among married women in Cameroon: Evidence from the 2018 Cameroon Demographic and Health Survey. J Biosoc Sci. 2021. 10.1017/S0021932021000079 33632369

[18] Agaba P, Magadi M, Onukwugha F, Misinde C. Factors associated with the timing and number of antenatal care visits among unmarried compared to married youth in Uganda between 2006 and 2016. Social Sciences. 2021;10(12).

[19] Afiaz A, Biswas RK. Awareness on menstrual hygiene management in Bangladesh and the possibilities of media interventions: using a nationwide cross-sectional survey. BMJ Open. 2021;11(4):e042134. doi: 10.1136/bmjopen-2020-042134 33858864 PMC8055124

[20] Saurman E. Improving access: modifying Penchansky and Thomas’s Theory of Access. J Health Serv Res Policy. 2016;21(1):36–9. doi: 10.1177/1355819615600001 26377728

[21] Penchansky R, Thomas JW. The concept of access: definition and relationship to consumer satisfaction. Med Care. 1981;19(2):127–40. doi: 10.1097/00005650-198102000-00001 7206846

[22] DHS. The Demographic and Health Surveys (DHS) Program [Internet]. [cited 2024 Jul 14]. Available from: https://dhsprogram.com/.

[23] Biswas RK, Farzana M, Bhar S, Bhowmik J. Contraceptive use in South and South-East Asian region: assessment of sustainable development goal 3.7 through indicator 3.7.1. J Public Health (Oxf). 2023;45(2):450–61. doi: 10.1093/pubmed/fdac105 36214514

[24] Vollmer S, Harttgen K, Kupka R, Subramanian S V. Levels and trends of childhood undernutrition by wealth and education according to a Composite Index of Anthropometric Failure: Evidence from 146 Demographic and Health Surveys from 39 countries. Vol. 2, BMJ Global Health. 2017. 10.1136/bmjgh-2016-000206 PMC5656130 29081994

[25] Hothorn T, Hornik K, Wien W, Zeileis A. ctree: Conditional Inference Trees. The Comprehensive R Archive Network. 2015;(Quinlan 1993):1–34.

[26] R Core Team. R: A language and environment for statistical computing. R Foundation for Statistical Computing, Vienna, Austria. Open J Stat. 2023;13(02).

[27] Wickham H, Averick M, Bryan J, Chang W, McGowan L, François R, et al. Welcome to the Tidyverse. JOSS. 2019;4(43):1686. doi: 10.21105/joss.01686

[28] Lumley T, Gao P, Schneider B. Package ‘survey’: Analysis of Complex Survey Samples. R package version 41. 2023

[29] Wickham H, Chang W, Wickham MH. Package `ggplot2’. Create Elegant Data Visualisations Using the Grammar of Graphics Version. 2016;2(1).

[30] Auguie B, Antonov A, Auguie MB, Robinson D. Package ‘gridExtra’. Miscellaneous Functions for “Grid” Graphics. 2017.

[31] Runfola D, Anderson A, Baier H, Crittenden M, Dowker E, Fuhrig S, et al. geoBoundaries: A global database of political administrative boundaries. PLoS One. 2020;15(4):e0231866. doi: 10.1371/journal.pone.0231866 32330167 PMC7182183

[32] Kutner M, Nachtsheim C, Neter J, Li W. Applied Statistical Linear Models. McGraw Hill. 2005.

[33] Nawaz F, Bushra AN. Health-Seeking Behavior of Rural Ethnic Women in Bangladesh: A critical analysis through intersectional lens. Arab Economic and Business Journal. 2023;15(2). doi: 10.38039/2214-4625.1029

[34] Stanikzai MH, Tawfiq E, Suwanbamrung C, Wasiq AW, Wongrith P. Predictors of antenatal care services utilization by pregnant women in Afghanistan: Evidence from the Afghanistan Health Survey 2018. PLoS One. 2024;19(10):e0309300. doi: 10.1371/journal.pone.0309300 39356654 PMC11446418

[35] Story WT, Burgard SA, Lori JR, Taleb F, Ali NA, Hoque DME. Husbands’ involvement in delivery care utilization in rural Bangladesh: A qualitative study. BMC Pregnancy Childbirth. 2012;12:28. doi: 10.1186/1471-2393-12-28 22494576 PMC3364886

[36] Kibria GMA, Nayeem J. Association of rural-urban place of residence with adequate antenatal care visit in Bangladesh. PLOS Glob Public Health. 2023;3(10):e0002528. doi: 10.1371/journal.pgph.0002528 37878558 PMC10599580

[37] Tran TK, Nguyen CTK, Nguyen HD, Eriksson B, Bondjers G, Gottvall K, et al. Urban - rural disparities in antenatal care utilization: a study of two cohorts of pregnant women in Vietnam. BMC Health Serv Res. 2011;11:120. doi: 10.1186/1472-6963-11-120 21605446 PMC3224373

[38] Tanou M, Kamiya Y. Assessing the impact of geographical access to health facilities on maternal healthcare utilization: Evidence from the Burkina Faso demographic and health survey 2010. BMC Public Health. 2019;19(1). 10.1186/s12889-019-7150-1 PMC6598277 31248393

[39] Tanou M, Kishida T, Kamiya Y. The effects of geographical accessibility to health facilities on antenatal care and delivery services utilization in Benin: a cross-sectional study. Reprod Health. 2021;18(1):205. doi: 10.1186/s12978-021-01249-x 34649581 PMC8518195

[40] Hinata H, Lwin KS, Eguchi A, Ghaznavi C, Hashizume M, Nomura S. Factors associated with barriers to healthcare access among ever-married women of reproductive age in Bangladesh: Analysis from the 2017-2018 Bangladesh Demographic and Health Survey. PLoS One. 2024;19(1):e0289324. doi: 10.1371/journal.pone.0289324 38181039 PMC10769052

[41] Nayem HM, Sinha A, Tareq R. Assessing the determinants of the frequency and contents of antenatal care visits in Bangladesh by using binary logistic regression. 2024. Available from: doi: 10.21203/rs.3.rs-4189149/v1

[42] Bhowmik J, Biswas R, Woldegiorgis M. Antenatal care and skilled birth attendance in Bangladesh are influenced by female education and family affordability: BDHS 2014. Public Health. 2019;170. 10.1016/j.puhe.2019.02.027 30991173

[43] Shibre G, Mekonnen W. Socio-economic inequalities in ANC attendance among mothers who gave birth in the past 12 months in Debre Brehan town and surrounding rural areas, North East Ethiopia: a community-based survey. Reprod Health. 2019;16(1):99. doi: 10.1186/s12978-019-0768-8 31286965 PMC6615209

[44] Mamuye Azanaw M, Gebremariam AD, Teshome Dagnaw F, Yisak H, Atikilt G, Minuye B, et al. Factors Associated with Numbers of Antenatal Care Visits in Rural Ethiopia. J Multidiscip Healthc. 2021;14:1403–11. doi: 10.2147/JMDH.S308802 34140778 PMC8203265

[45] Hossain M, Rahman T, Sadia T, Saleheen A, Sarkar S, Khan M, et al. Survival analysis of early intention of antenatal care among women in Bangladesh. Scientific Reports. n.d.;14(1):PAGE_RANGE. doi: DOI_NUMBER 10.1038/s41598-024-55443-5 PMC10899645 38413798

[46] Awoke N, Ababulgu SA, Hanfore LK, Gebeyehu EG, Wake SK. Regional disparities in antenatal care utilization among pregnant women and its determinants in Ethiopia. Front Glob Womens Health. 2024;5:1230975. doi: 10.3389/fgwh.2024.1230975 38404954 PMC10884275

[47] Laksono AD, Rukmini R, Wulandari RD. Regional disparities in antenatal care utilization in Indonesia. PLoS One. 2020;15(2):e0224006. doi: 10.1371/journal.pone.0224006 32053621 PMC7018075

[48] Mohammad KA, Zahura FT, Rahman MM. Importance of maternal education on antenatal care visits in Bangladesh. Bangladesh J Sci Res. 2018;30(1–2):23–33. doi: 10.3329/bjsr.v30i1-2.36117

[49] Bhowmik J, Biswas RK, Ananna N. Women’s education and coverage of skilled birth attendance: An assessment of Sustainable Development Goal 3.1 in the South and Southeast Asian Region. PLoS One. 2020;15(4):e0231489. doi: 10.1371/journal.pone.0231489 32315328 PMC7173780

[50] Fagbamigbe A, Mashabe B, Lepetu L, Abel C. Are the timings and risk factors changing? Survival analysis of timing of first antenatal care visit among pregnant women in Nigeria (2003-2013). Int J Womens Health. 2017;9. 10.2147/IJWH.S138329 PMC5669794 29133984

[51] Adebangbe FT, Mturi AJ. Factors associated with the number of antenatal care visits among internally displaced women in northern Nigeria. Afr J Reprod Health. 2021;25(2). 10.29063/ajrh2021/v25i2.12 37585760

[52] Pickard A, Islam MI, Ahmed MS, Martiniuk A. Role of internet use, mobile phone, media exposure and domestic migration on reproductive health service use in Bangladeshi married adolescents and young women. PLOS Glob Public Health. 2024;4(3):e0002518. doi: 10.1371/journal.pgph.0002518 38437231 PMC10911608

[53] Yaya S, Bishwajit G, Ekholuenetale M, Shah V, Kadio B, Udenigwe O. Timing and adequate attendance of antenatal care visits among women in Ethiopia. PLoS One. 2017;12(9):e0184934. doi: 10.1371/journal.pone.0184934 28922383 PMC5602662

[54] Manyeh AK, Amu A, Williams J, Gyapong M. Factors associated with the timing of antenatal clinic attendance among first-time mothers in rural southern Ghana. BMC Pregnancy Childbirth. 2020;20(1):47. doi: 10.1186/s12884-020-2738-0 31959137 PMC6972022

[55] Daniels-Donkor S, Afaya A, Daliri D, Laari T, Salia S, Avane M. Factors associated with timely initiation of antenatal care among reproductive age women in The Gambia: a multilevel fixed effects analysis. Archives of Public Health. 2024;82(1). 10.1186/s13690-024-01247-y PMC11100154 38760806

[56] Thorsteinsen K, Parks-Stamm EJ, Kvalø M, Olsen M, Martiny SE. Mothers’ Domestic Responsibilities and Well-Being During the COVID-19 Lockdown: The Moderating Role of Gender Essentialist Beliefs About Parenthood. Sex Roles. 2022;87(1–2):85–98. doi: 10.1007/s11199-022-01307-z 35813971 PMC9253260

[57] Makate M. Maternal health-seeking behavior and child’s birth order: Evidence from Malawi, Uganda, and Zimbabwe. Munich Personal RePEc Archive. 2016;(72722).

[58] Muchie KF. Quality of antenatal care services and completion of four or more antenatal care visits in Ethiopia: A finding based on a demographic and health survey. BMC Pregnancy Childbirth. 2017;17(1). 10.1186/s12884-017-1488-0 PMC5594613 28893222

