# Supplementary material for: More than just visits: Timing, frequency, and determinants of effective antenatal care in Bangladesh - BDHS 2007 to 2017-18
Source: PLoS One. 2025 May 2;20(5):e0321686. doi: 10.1371/journal.pone.0321686 (PMC12047838; doi:10.1371/journal.pone.0321686)
Supplement: S1 Table — (DOCX) [file pone.0321686.s001.docx]

S1 Table: The frequency distribution of late ANC and low (< 8) ANC visits by sociodemographic factors.

| **Characteristic** | **BDHS 2007** | | | **BDHS 2017-18** | | |
| --- | --- | --- | --- | --- | --- | --- |
|  | **Total sample,**  **N = 2,949** | **N (%) of women with Late ANC visits** | **N (%) of women with Low (< 8) ANC**  **visits** | **Total sample,**  **N = 4,588 (%)** | **N (%) of women with Late ANC visits** | **N (%) of women with Low (< 8) ANC**  **visits** |
| **Area of residence** |  |  |  |  |  |  |
| Urban | 783 (26.5) | 379 (48.4) | 659 (84.2) | 1,266 (27.6) | 634 (50.0) | 1,041 (82.2) |
| Rural | 2,166 (73.5) | 1,377 (63.6) | 2,077 (95.9) | 3,322 (72.4) | 2,104 (63.3) | 2,995 (90.1) |
| **Wealth index** |  |  |  |  |  |  |
| Poorest | 446 (15.1) | 325 (72.9) | 441 (99.1) | 848 (18.5) | 620 (73.2) | 797 (94.0) |
| Poorer | 495 (16.8) | 339 (68.5) | 488 (98.5) | 912 (19.9) | 605 (66.3) | 832 (91.2) |
| Middle | 549 (18.6) | 354 (64.6) | 530 (96.6) | 894 (19.5) | 561 (62.8) | 795 (89.0) |
| Richer | 688 (23.3) | 397 (57.8) | 644 (93.7) | 970 (21.2) | 582 (60.0) | 861 (88.8) |
| Richest | 772 (26.2) | 340 (44.1) | 632 (81.9) | 964 (21.0) | 369 (38.2) | 751 (77.8) |
| **Region** |  |  |  |  |  |  |
| Dhaka | 882 (29.9) | 505 (57.3) | 803 (91.0) | 1,185 (25.8) | 595 (50.2) | 998 (84.2) |
| Barishal | 164 (5.6) | 95 (57.6) | 155 (94.5) | 241 (5.3) | 155 (64.2) | 213 (88.1) |
| Chattogram | 617 (20.9) | 343 (55.5) | 565 (91.6) | 966 (21.1) | 633 (65.5) | 893 (92.4) |
| Khulna | 356 (12.1) | 214 (60.1) | 324 (90.9) | 439 (9.6) | 263 (60.0) | 383 (87.4) |
| Mymensingh |  |  |  | 381 (8.3) | 228 (59.8) | 337 (88.6) |
| Rajshahi | 721 (24.4) | 479 (66.4) | 689 (95.6) | 550 (12.0) | 366 (66.4) | 478 (86.8) |
| Rangpur |  |  |  | 502 (10.9) | 322 (64.0) | 431 (85.8) |
| Sylhet | 209 (7.1) | 121 (58.1) | 200 (95.8) | 323 (7.0) | 176 (54.4) | 302 (93.3) |
| **Women's age (Mean [SD])** | 24.9 (5.8) | 24.5 (5.8) | 24.8 (5.9) | 24.8 (5.5) | 24.7 (5.6) | 24.7 (5.5) |
| **Women’s education level** |  |  |  |  |  |  |
| No education | 474 (16.1) | 311 (65.7) | 466 (98.3) | 229 (5.0) | 166 (72.7) | 221 (96.6) |
| Primary | 828 (28.1) | 541 (65.3) | 798 (96.3) | 1,198 (26.1) | 841 (70.2) | 1,102 (91.9) |
| Secondary | 1,340 (45.4) | 787 (58.8) | 1,235 (92.2) | 2,314 (50.4) | 1,404 (60.7) | 2,049 (88.5) |
| Higher | 307 (10.4) | 117 (38.0) | 237 (77.3) | 847 (18.5) | 326 (38.5) | 664 (78.4) |
| **Women’s employment status** |  |  |  |  |  |  |
| Not working | 2,193 (74.4) | 1,293 (58.9) | 2,017 (92.0) | 2,919 (63.6) | 1,653 (56.6) | 2,562 (87.8) |
| Working | 756 (25.6) | 464 (61.3) | 718 (95.0) | 1,669 (36.4) | 1,084 (64.9) | 1,473 (88.3) |
| **Partner’s education level** |  |  |  |  |  |  |
| No education | 734 (24.9) | 516 (70.3) | 715 (97.4) | 576 (12.5) | 421 (73.2) | 536 (93.1) |
| Primary | 775 (26.3) | 483 (62.3) | 745 (96.1) | 1,488 (32.4) | 1,025 (68.9) | 1,374 (92.4) |
| Secondary | 964 (32.7) | 562 (58.3) | 889 (92.3) | 1,617 (35.3) | 944 (58.4) | 1,416 (87.5) |
| Higher | 476 (16.1) | 195 (41.0) | 386 (81.1) | 907 (19.8) | 347 (38.2) | 709 (78.2) |
| **Media exposure** |  |  |  |  |  |  |
| No | 795 (27.0) | 532 (66.9) | 779 (98.0) | 1,458 (31.8) | 1,021 (70.0) | 1,368 (93.8) |
| Yes | 2,154 (73.0) | 1,225 (56.9) | 1,957 (90.9) | 3,130 (68.2) | 1,717 (54.8) | 2,667 (85.2) |
| **Birth order** |  |  |  |  |  |  |
| 1 | 1,162 (39.4) | 669 (57.6) | 1,064 (91.5) | 1,815 (39.6) | 1,007 (55.5) | 1,564 (86.2) |
| 2-3 | 1,298 (44.0) | 795 (61.3) | 1,196 (92.2) | 2,276 (49.6) | 1,373 (60.3) | 1,994 (87.6) |
| 4+ | 489 (16.6) | 292 (59.7) | 476 (97.3) | 498 (10.8) | 358 (71.9) | 477 (95.8) |
| **Distance to health facility** |  |  |  |  |  |  |
| Not a big problem |  |  |  | 2,758 (60.1) | 1,563 (56.7) | 2,370 (85.9) |
| Big problem |  |  |  | 1,830 (39.9) | 1,174 (64.1) | 1,665 (91.0) |
| **Owning mobile phone** |  |  |  |  |  |  |
| No |  |  |  | 1,670 (36.4) | 1,136 (68.0) | 1,534 (91.8) |
| Yes |  |  |  | 2,918 (63.6) | 1,602 (54.9) | 2,501 (85.7) |
