# Supplementary material for: More than just visits: Timing, frequency, and determinants of effective antenatal care in Bangladesh - BDHS 2007 to 2017-18
Source: PLoS One. 2025 May 2;20(5):e0321686. doi: 10.1371/journal.pone.0321686 (PMC12047838; doi:10.1371/journal.pone.0321686)
Supplement: S2 Table — (DOCX) [file pone.0321686.s002.docx]

S2 Table: The frequency distribution of low (<8) ANC visits by timing of first ANC visits.

| **Characteristic** | **BDHS 2007** | | **BDHS 2017-18** | |
| --- | --- | --- | --- | --- |
|  | **Total sample,**  **N = 2,949** | **N (%) of women with Low (< 8) ANC**  **visits** | **Total sample,**  **N = 4,588 (%)** | **N (%) of women with Low (< 8) ANC**  **visits** |
| **Timing of first ANC visit** |  |  |  |  |
| Not late | 1,193 (40.4) | 1,000 (83.9) | 1,851 (40.3) | 1,403 (75.8) |
| Late | 1,756 (59.6) | 1,735 (98.8) | 2,737 (59.7) | 2,632 (96.2) |
