# Supplementary material for: More than just visits: Timing, frequency, and determinants of effective antenatal care in Bangladesh - BDHS 2007 to 2017-18
Source: PLoS One. 2025 May 2;20(5):e0321686. doi: 10.1371/journal.pone.0321686 (PMC12047838; doi:10.1371/journal.pone.0321686)
Supplement: S3 Table — (DOCX) [file pone.0321686.s003.docx]

S3 Table: Binary logistic regression model adjusted for sociodemographic factors and timing of first ANC visit with number of ANC visits (low (<4) ANC visits) as outcome.

| **Characteristic** | **BDHS 2007** | | | **BDHS 2017-18** | | |
| --- | --- | --- | --- | --- | --- | --- |
|  | **AOR** | **95% CI** | **p-value** | **AOR** | **95% CI** | **p-value** |
| **Timing of first ANC visit** |  |  |  |  |  |  |
| Not late (ref.) | — | — |  | — | — |  |
| Late | 4.60 | 3.69, 5.73 | **<0.001** | 4.68 | 4.00, 5.48 | **<0.001** |
| **Area of residence** |  |  |  |  |  |  |
| Urban (ref.) | — | — |  | — | — |  |
| Rural | 1.65 | 1.32, 2.07 | **<0.001** | 1.29 | 1.06, 1.56 | **0.011** |
| **Wealth index** |  |  |  |  |  |  |
| Poorest (ref.) | — | — |  | — | — |  |
| Poorer | 1.09 | 0.69, 1.70 | 0.714 | 1.10 | 0.86, 1.42 | 0.448 |
| Middle | 1.01 | 0.66, 1.54 | 0.976 | 0.87 | 0.67, 1.13 | 0.295 |
| Richer | 0.75 | 0.49, 1.15 | 0.189 | 0.76 | 0.58, 1.00 | 0.053 |
| Richest | 0.57 | 0.35, 0.94 | **0.028** | 0.52 | 0.37, 0.71 | **<0.001** |
| **Region** |  |  |  |  |  |  |
| Dhaka (ref.) | — | — |  | — | — |  |
| Barishal | 0.91 | 0.63, 1.30 | 0.601 | 0.93 | 0.69, 1.24 | 0.612 |
| Chattogram | 1.138 | 0.86, 1.50 | 0.381 | 1.22 | 0.94, 1.57 | 0.138 |
| Khulna | 0.86 | 0.63, 1.17 | 0.332 | 0.58 | 0.43, 0.78 | **<0.001** |
| Mymensingh |  |  |  | 0.73 | 0.54, 0.99 | **0.040** |
| Rajshahi | 0.56 | 0.41, 0.78 | **<0.001** | 0.76 | 0.58, 1.01 | 0.059 |
| Rangpur |  |  |  | 0.36 | 0.27, 0.49 | **<0.001** |
| Sylhet | 1.44 | 0.95, 2.19 | 0.089 | 1.21 | 0.92, 1.59 | 0.174 |
| **Women's age** | 0.98 | 0.95, 1.00 | 0.092 | 0.97 | 0.95, 0.99 | **0.003** |
| **Women’s education level** |  |  |  |  |  |  |
| No education (ref.) | — | — |  | — | — |  |
| Primary | 0.87 | 0.59, 1.29 | 0.490 | 0.59 | 0.40, 0.87 | **0.009** |
| Secondary | 0.54 | 0.36, 0.81 | **0.003** | 0.49 | 0.33, 0.72 | **<0.001** |
| Higher | 0.36 | 0.21, 0.61 | **<0.001** | 0.53 | 0.33, 0.83 | **0.006** |
| **Women’s employment status** |  |  |  |  |  |  |
| Not working (ref.) | — | — |  | — | — |  |
| Working | 1.06 | 0.83, 1.34 | 0.656 | 0.81 | 0.69, 0.96 | **0.013** |
| **Partner’s education level** |  |  |  |  |  |  |
| No education (ref.) | — | — |  | — | — |  |
| Primary | 0.97 | 0.71, 1.33 | 0.841 | 1.01 | 0.79, 1.30 | 0.909 |
| Secondary | 0.81 | 0.59, 1.12 | 0.210 | 0.89 | 0.69, 1.16 | 0.386 |
| Higher | 0.64 | 0.43, 0.96 | **0.032** | 0.64 | 0.46, 0.89 | **0.008** |
| **Media exposure** |  |  |  |  |  |  |
| No (ref.) | — | — |  | — | — |  |
| Yes | 1.00 | 0.75, 1.34 | 0.981 | 0.75 | 0.62, 0.90 | **0.002** |
| **Birth order** |  |  |  |  |  |  |
| 1 (ref.) | — | — |  | — | — |  |
| 2-3 | 1.16 | 0.90, 1.48 | 0.249 | 1.29 | 1.06, 1.57 | **0.010** |
| 4+ | 1.93 | 1.22, 3.07 | **0.005** | 2.18 | 1.52, 3.12 | **<0.001** |
| **Distance to health facility** |  |  |  |  |  |  |
| Not a big problem (ref.) |  |  |  | — | — |  |
| Big problem |  |  |  | 1.18 | 1.01, 1.37 | **0.034** |
| **Owning mobile phone** |  |  |  |  |  |  |
| No (ref.) |  |  |  | — | — |  |
| Yes |  |  |  | 0.80 | 0.68, 0.94 | **0.006** |
| AOR = Adjusted Odds Ratio, CI = Confidence Interval | | | | | | |
