# Supplementary material for: More than just visits: Timing, frequency, and determinants of effective antenatal care in Bangladesh - BDHS 2007 to 2017-18
Source: PLoS One. 2025 May 2;20(5):e0321686. doi: 10.1371/journal.pone.0321686 (PMC12047838; doi:10.1371/journal.pone.0321686)
Supplement: S4 Table — (DOCX) [file pone.0321686.s004.docx]

S4 Table: Binary logistic regression model adjusted for sociodemographic factors with timing of first ANC visit and number of ANC visits (low (<8) ANC visits) as outcome.

| **Variables** | **Late ANC visits** | | | | | | **Low (< 8) ANC visits** | | | | | |
| --- | --- | --- | --- | --- | --- | --- | --- | --- | --- | --- | --- | --- |
|  | **BDHS 2007** | | | **BDHS 2017-18** | | | **BDHS 2007** | | | **BDHS 2017-18** | | |
|  | **AOR** | **95% CI** | **p-value** | **AOR** | **95% CI** | **p-value** | **AOR** | **95% CI** | **p-value** | **AOR** | **95% CI** | **p-value** |
| **Area of residence** |  |  |  |  |  |  |  |  |  |  |  |  |
| Urban (ref.) | — | — |  | — | — |  | — | — |  | — | — |  |
| Rural | 1.26 | 1.03, 1.53 | **0.023** | 1.14 | 0.97, 1.35 | 0.122 | 1.95 | 1.40, 2.71 | **<0.001** | 1.29 | 1.01, 1.64 | **0.044** |
| **Wealth index** |  |  |  |  |  |  |  |  |  |  |  |  |
| Poorest (ref.) | — | — |  | — | — |  | — | — |  | — | — |  |
| Poorer | 0.85 | 0.60, 1.20 | 0.358 | 0.88 | 0.69, 1.12 | 0.296 | 0.75 | 0.19, 2.98 | 0.686 | 0.87 | 0.58, 1.32 | 0.52 |
| Middle | 0.78 | 0.56, 1.09 | 0.14 | 0.91 | 0.71, 1.18 | 0.489 | 0.43 | 0.12, 1.54 | 0.196 | 0.8 | 0.52, 1.24 | 0.318 |
| Richer | 0.66 | 0.47, 0.92 | **0.014** | 1 | 0.76, 1.31 | 0.981 | 0.32 | 0.09, 1.11 | 0.072 | 0.96 | 0.62, 1.47 | 0.841 |
| Richest | 0.49 | 0.34, 0.72 | **<0.001** | 0.58 | 0.43, 0.79 | **<0.001** | 0.18 | 0.05, 0.60 | **0.005** | 0.6 | 0.38, 0.96 | **0.034** |
| **Region** |  |  |  |  |  |  |  |  |  |  |  |  |
| Dhaka (ref.) | — | — |  | — | — |  | — | — |  | — | — |  |
| Barishal | 0.83 | 0.62, 1.13 | 0.238 | 1.4 | 1.07, 1.82 | **0.013** | 1.02 | 0.61, 1.72 | 0.927 | 0.95 | 0.63, 1.43 | 0.792 |
| Chattogram | 0.93 | 0.73, 1.19 | 0.556 | 1.73 | 1.38, 2.17 | **<0.001** | 0.89 | 0.61, 1.30 | 0.545 | 1.87 | 1.31, 2.66 | **<0.001** |
| Khulna | 1.03 | 0.77, 1.37 | 0.845 | 1.29 | 0.99, 1.68 | 0.063 | 0.76 | 0.49, 1.18 | 0.221 | 1.11 | 0.78, 1.58 | 0.578 |
| Mymensingh |  |  |  | 1.09 | 0.82, 1.44 | 0.563 |  |  |  | 1.01 | 0.69, 1.48 | 0.966 |
| Rajshahi | 1.25 | 0.96, 1.63 | 0.093 | 1.63 | 1.24, 2.14 | **<0.001** | 1.4 | 0.86, 2.29 | 0.174 | 0.98 | 0.68, 1.43 | 0.936 |
| Rangpur |  |  |  | 1.32 | 0.99, 1.74 | 0.055 |  |  |  | 0.78 | 0.53, 1.15 | 0.204 |
| Sylhet | 0.89 | 0.63, 1.25 | 0.508 | 0.79 | 0.61, 1.02 | 0.071 | 1.31 | 0.62, 2.80 | 0.482 | 1.64 | 1.00, 2.70 | **0.049** |
| **Women's age** | 0.97 | 0.95, 0.99 | **0.003** | 0.98 | 0.96, 1.00 | **0.036** | 0.95 | 0.91, 0.99 | **0.012** | 0.95 | 0.93, 0.97 | **<0.001** |
| **Women’s education level** |  |  |  |  |  |  |  |  |  |  |  |  |
| No education (ref.) | — | — |  | — | — |  | — | — |  | — | — |  |
| Primary | 1.04 | 0.78, 1.40 | 0.787 | 0.93 | 0.65, 1.34 | 0.702 | 0.63 | 0.27, 1.49 | 0.291 | 0.42 | 0.18, 0.99 | **0.046** |
| Secondary | 0.98 | 0.71, 1.36 | 0.911 | 0.76 | 0.53, 1.09 | 0.13 | 0.45 | 0.19, 1.07 | 0.07 | 0.39 | 0.17, 0.90 | **0.027** |
| Higher | 0.77 | 0.49, 1.20 | 0.25 | 0.52 | 0.35, 0.78 | **0.002** | 0.34 | 0.13, 0.86 | **0.023** | 0.34 | 0.14, 0.82 | **0.016** |
| **Women’s employment status** |  |  |  |  |  |  |  |  |  |  |  |  |
| Not working (ref.) | — | — |  | — | — |  | — | — |  | — | — |  |
| Working | 0.93 | 0.76, 1.15 | 0.524 | 1.08 | 0.94, 1.26 | 0.281 | 1.3 | 0.87, 1.97 | 0.205 | 0.89 | 0.70, 1.12 | 0.312 |
| **Partner’s education level** |  |  |  |  |  |  |  |  |  |  |  |  |
| No education (ref.) | — | — |  | — | — |  | — | — |  | — | — |  |
| Primary | 0.75 | 0.58, 0.97 | 0.03 | 0.92 | 0.71, 1.20 | 0.558 | 1.12 | 0.55, 2.27 | 0.763 | 1.12 | 0.74, 1.69 | 0.598 |
| Secondary | 0.79 | 0.60, 1.04 | 0.098 | 0.73 | 0.56, 0.96 | **0.023** | 1.02 | 0.53, 1.98 | 0.947 | 0.81 | 0.53, 1.23 | 0.323 |
| Higher | 0.52 | 0.36, 0.76 | **<0.001** | 0.49 | 0.36, 0.68 | **<0.001** | 0.7 | 0.34, 1.41 | 0.314 | 0.68 | 0.42, 1.09 | 0.105 |
| **Media exposure** |  |  |  |  |  |  |  |  |  |  |  |  |
| No (ref.) | — | — |  | — | — |  | — | — | — | — |  | — |
| Yes | 0.98 | 0.77, 1.23 | 0.848 | 0.79 | 0.67, 0.94 | **0.008** | 0.69 | 0.37, 1.26 | 0.62 | 0.46, 0.84 | **0.002** | 0.62 |
| **Birth order** |  |  |  |  |  |  |  |  |  |  |  |  |
| 1 (ref.) | — | — |  | — | — |  | — | — | — | — |  | — |
| 2-3 | 1.29 | 1.04, 1.60 | **0.022** | 1.17 | 0.97, 1.42 | 0.097 | 1.26 | 0.86, 1.85 | 1.38 | 1.06, 1.79 | **0.016** | 1.38 |
| 4+ | 1.19 | 0.82, 1.72 | 0.357 | 1.55 | 1.08, 2.24 | **0.018** | 2.8 | 1.25, 6.27 | 3.89 | 2.09, 7.23 | **<0.001** | 3.89 |
| **Distance to health facility** |  |  |  |  |  |  |  |  |  |  |  |  |
| Not a big problem (ref.) |  |  |  | — | — |  |  |  | — | — |  | — |
| Big problem |  |  |  | 1.1 | 0.94, 1.27 | 0.233 |  |  | 1.23 | 0.98, 1.53 | 0.07 | 1.23 |
| **Owning mobile phone** |  |  |  |  |  |  |  |  |  |  |  |  |
| No (ref.) |  |  |  | — | — |  |  |  | — | — |  | — |
| Yes |  |  |  | 0.84 | 0.72, 0.97 | **0.021** |  |  | 0.81 | 0.63, 1.04 | 0.096 | 0.81 |
| AOR = Adjusted Odds Ratio, CI = Confidence Interval | | | | | | | | | | | | |
