# Supplementary material for: More than just visits: Timing, frequency, and determinants of effective antenatal care in Bangladesh - BDHS 2007 to 2017-18
Source: PLoS One. 2025 May 2;20(5):e0321686. doi: 10.1371/journal.pone.0321686 (PMC12047838; doi:10.1371/journal.pone.0321686)
Supplement: S5 Table — (DOCX) [file pone.0321686.s005.docx]

S5 Table: Binary logistic regression model adjusted for sociodemographic factors and timing of first ANC visit with number of ANC visits (low (<8) ANC visits) as outcome.

| **Characteristic** | **BDHS 2007** | | | **BDHS 2017-18** | | |
| --- | --- | --- | --- | --- | --- | --- |
|  | **AOR** | **95% CI** | **p-value** | **AOR** | **95% CI** | **p-value** |
| **Timing of first ANC visit** |  |  |  |  |  |  |
| Not late (ref.) | — | — |  | — | — |  |
| Late | 11.2 | 6.83, 18.3 | **<0.001** | 6.43 | 4.99, 8.28 | **<0.001** |
| **Area of residence** |  |  |  |  |  |  |
| Urban (ref.) | — | — |  | — | — |  |
| Rural | 1.71 | 1.19, 2.46 | **0.004** | 1.22 | 0.94, 1.58 | 0.127 |
| **Wealth index** |  |  |  |  |  |  |
| Poorest (ref.) | — | — |  | — | — |  |
| Poorer | 0.8 | 0.20, 3.28 | 0.758 | 0.91 | 0.59, 1.39 | 0.659 |
| Middle | 0.48 | 0.13, 1.78 | 0.271 | 0.81 | 0.51, 1.28 | 0.369 |
| Richer | 0.39 | 0.11, 1.42 | 0.155 | 0.97 | 0.61, 1.52 | 0.886 |
| Richest | 0.23 | 0.06, 0.82 | **0.023** | 0.71 | 0.43, 1.19 | 0.194 |
| **Region** |  |  |  |  |  |  |
| Dhaka (ref.) | — | — |  | — | — |  |
| Barishal | 1.04 | 0.61, 1.80 | 0.875 | 0.83 | 0.54, 1.28 | 0.397 |
| Chattogram | 0.87 | 0.58, 1.32 | 0.519 | 1.56 | 1.08, 2.26 | **0.019** |
| Khulna | 0.7 | 0.44, 1.13 | 0.149 | 0.99 | 0.68, 1.43 | 0.944 |
| Mymensingh |  |  |  | 0.97 | 0.65, 1.44 | 0.872 |
| Rajshahi | 1.2 | 0.70, 2.05 | 0.51 | 0.82 | 0.55, 1.21 | 0.313 |
| Rangpur |  |  |  | 0.67 | 0.45, 1.01 | 0.054 |
| Sylhet | 1.52 | 0.70, 3.30 | 0.287 | 1.85 | 1.11, 3.06 | **0.017** |
| **Women's age** | 0.96 | 0.91, 1.00 | 0.052 | 0.95 | 0.93, 0.98 | **<0.001** |
| **Women’s education level** |  |  |  |  |  |  |
| No education (ref.) | — | — |  | — | — |  |
| Primary | 0.68 | 0.28, 1.69 | 0.411 | 0.4 | 0.16, 0.98 | **0.045** |
| Secondary | 0.51 | 0.20, 1.26 | 0.142 | 0.41 | 0.17, 0.97 | **0.042** |
| Higher | 0.41 | 0.15, 1.11 | 0.081 | 0.4 | 0.16, 1.00 | **0.049** |
| **Women’s employment status** |  |  |  |  |  |  |
| Not working (ref.) | — | — |  | — | — |  |
| Working | 1.3 | 0.83, 2.04 | 0.247 | 0.85 | 0.66, 1.09 | 0.193 |
| **Partner’s education level** |  |  |  |  |  |  |
| No education (ref.) | — | — |  | — | — |  |
| Primary | 1.27 | 0.61, 2.66 | 0.526 | 1.13 | 0.73, 1.74 | 0.587 |
| Secondary | 1.13 | 0.57, 2.23 | 0.731 | 0.92 | 0.59, 1.44 | 0.718 |
| Higher | 0.88 | 0.43, 1.82 | 0.735 | 0.86 | 0.52, 1.43 | 0.558 |
| **Media exposure** |  |  |  |  |  |  |
| No (ref.) | — | — |  | — | — |  |
| Yes | 0.69 | 0.36, 1.30 | 0.25 | 0.68 | 0.49, 0.92 | **0.014** |
| **Birth order** |  |  |  |  |  |  |
| 1 (ref.) | — | — |  | — | — |  |
| 2-3 | 1.09 | 0.72, 1.65 | 0.67 | 1.31 | 1.00, 1.72 | 0.051 |
| 4+ | 2.8 | 1.15, 6.81 | **0.023** | 3.36 | 1.78, 6.34 | **<0.001** |
| **Distance to health facility** |  |  |  |  |  |  |
| Not a big problem (ref.) |  |  |  | — | — |  |
| Big problem |  |  |  | 1.22 | 0.97, 1.53 | 0.088 |
| **Owning mobile phone** |  |  |  |  |  |  |
| No (ref.) |  |  |  | — | — |  |
| Yes |  |  |  | 0.87 | 0.67, 1.12 | 0.28 |
| AOR = Adjusted Odds Ratio, CI = Confidence Interval | | | | | | |
