# Supplementary material for: More than just visits: Timing, frequency, and determinants of effective antenatal care in Bangladesh - BDHS 2007 to 2017-18
Source: PLoS One. 2025 May 2;20(5):e0321686. doi: 10.1371/journal.pone.0321686 (PMC12047838; doi:10.1371/journal.pone.0321686)
Supplement: S6 Table — (DOCX) [file pone.0321686.s006.docx]

S6 Table: GVIF for binary logistic regression model adjusted for sociodemographic factors with timing of first ANC visit as outcome.

|  | **BDHS 2007** | | | | **BDHS 2017-18** | | | |
| --- | --- | --- | --- | --- | --- | --- | --- | --- |
| **Characteristic** | **GVIF** | **Df** | **Adjusted GVIF** | **Squared Adjusted GVIF** | **GVIF** | **Df** | **Adjusted GVIF** | **Squared Adjusted GVIF** |
| **Area of residence** | 1.51 | 1 | 1.23 | 1.51 | 1.43 | 1 | 1.19 | 1.42 |
| **Wealth index** | 2.85 | 4 | 1.14 | 1.30 | 2.40 | 4 | 1.12 | 1.25 |
| **Region** | 1.42 | 5 | 1.04 | 1.08 | 1.72 | 7 | 1.04 | 1.08 |
| **Women's age** | 2.13 | 1 | 1.46 | 2.13 | 1.98 | 1 | 1.41 | 1.99 |
| **Women’s education level** | 2.80 | 3 | 1.19 | 1.42 | 2.18 | 3 | 1.14 | 1.30 |
| **Women’s employment status** | 1.08 | 1 | 1.04 | 1.08 | 1.15 | 1 | 1.07 | 1.14 |
| **Partner’s education level** | 2.53 | 3 | 1.17 | 1.37 | 2.08 | 3 | 1.13 | 1.28 |
| **Media exposure** | 1.46 | 1 | 1.21 | 1.46 | 1.33 | 1 | 1.15 | 1.32 |
| **Birth order** | 2.39 | 2 | 1.24 | 1.54 | 1.06 | 2 | 1.03 | 1.06 |
| **Distance to health facility** |  |  |  |  | 2.23 | 1 | 1.22 | 1.49 |
| **Owning mobile phone** |  |  |  |  | 1.18 | 1 | 1.09 | 1.19 |
