# Supplementary material for: More than just visits: Timing, frequency, and determinants of effective antenatal care in Bangladesh - BDHS 2007 to 2017-18
Source: PLoS One. 2025 May 2;20(5):e0321686. doi: 10.1371/journal.pone.0321686 (PMC12047838; doi:10.1371/journal.pone.0321686)
Supplement: S7 Table — (DOCX) [file pone.0321686.s007.docx]

S7 Table: GVIF for binary logistic regression model adjusted for sociodemographic factors with number of ANC visits (low (<4) ANC visits) as outcome.

|  | **BDHS 2007** | | | | **BDHS 2017-18** | | | |
| --- | --- | --- | --- | --- | --- | --- | --- | --- |
| **Characteristic** | **GVIF** | **Df** | **Adjusted GVIF** | **Squared Adjusted GVIF** | **GVIF** | **Df** | **Adjusted GVIF** | **Squared Adjusted GVIF** |
| **Area of residence** | 1.45 | 1 | 1.20 | 1.44 | 1.41 | 1 | 1.19 | 1.42 |
| **Wealth index** | 2.83 | 4 | 1.14 | 1.30 | 2.43 | 4 | 1.03 | 1.06 |
| **Region** | 1.44 | 5 | 1.04 | 1.08 | 1.60 | 7 | 1.03 | 1.06 |
| **Women's age** | 2.21 | 1 | 1.49 | 2.22 | 1.99 | 1 | 1.41 | 1.99 |
| **Women’s education level** | 2.63 | 3 | 1.18 | 1.39 | 1.91 | 3 | 1.11 | 1.23 |
| **Women’s employment status** | 1.09 | 1 | 1.05 | 1.10 | 1.24 | 1 | 1.12 | 1.25 |
| **Partner’s education level** | 2.06 | 3 | 1.13 | 1.28 | 1.98 | 3 | 1.12 | 1.25 |
| **Media exposure** | 1.47 | 1 | 1.21 | 1.46 | 1.38 | 1 | 1.18 | 1.39 |
| **Birth order** | 2.27 | 2 | 1.23 | 1.51 | 2.12 | 2 | 1.21 | 1.46 |
| **Distance to health facility** |  |  |  |  | 1.08 | 1 | 1.04 | 1.08 |
| **Owning mobile phone** |  |  |  |  | 1.15 | 1 | 1.07 | 1.14 |
