# Supplementary material for: More than just visits: Timing, frequency, and determinants of effective antenatal care in Bangladesh - BDHS 2007 to 2017-18
Source: PLoS One. 2025 May 2;20(5):e0321686. doi: 10.1371/journal.pone.0321686 (PMC12047838; doi:10.1371/journal.pone.0321686)
Supplement: S8 Table — (DOCX) [file pone.0321686.s008.docx]

S8 Table: GVIF for binary logistic regression model adjusted for sociodemographic factors and timing of first ANC visit with number of ANC visits (low (<4) ANC visits) as outcome.

|  | **BDHS 2007** | | | | **BDHS 2017-18** | | | |
| --- | --- | --- | --- | --- | --- | --- | --- | --- |
| **Characteristic** | **GVIF** | **Df** | **Adjusted GVIF** | **Squared Adjusted GVIF** | **GVIF** | **Df** | **Adjusted GVIF** | **Squared Adjusted GVIF** |
| **Timing of ANC visit** | 1.15 | 1 | 1.07 | 1.14 | 1.08 | 1 | 1.04 | 1.08 |
| **Area of residence** | 1.49 | 1 | 1.22 | 1.49 | 1.41 | 1 | 1.19 | 1.42 |
| **Wealth index** | 3.24 | 4 | 1.16 | 1.35 | 2.37 | 4 | 1.11 | 1.23 |
| **Region** | 1.50 | 5 | 1.04 | 1.08 | 1.77 | 7 | 1.04 | 1.08 |
| **Women's age** | 2.46 | 1 | 1.57 | 2.46 | 1.98 | 1 | 1.41 | 1.99 |
| **Women’s education level** | 3.05 | 3 | 1.20 | 1.44 | 1.84 | 3 | 1.11 | 1.23 |
| **Women’s employment status** | 1.10 | 1 | 1.05 | 1.10 | 1.26 | 1 | 1.12 | 1.25 |
| **Partner’s education level** | 2.38 | 3 | 1.16 | 1.35 | 1.94 | 3 | 1.12 | 1.25 |
| **Media exposure** | 1.58 | 1 | 1.26 | 1.59 | 1.38 | 1 | 1.17 | 1.37 |
| **Birth order** | 2.56 | 2 | 1.27 | 1.61 | 1.09 | 2 | 1.05 | 1.10 |
| **Distance to health facility** |  |  |  |  | 2.16 | 1 | 1.21 | 1.46 |
| **Owning mobile phone** |  |  |  |  | 1.16 | 1 | 1.08 | 1.17 |
