# Supplementary material for: More than just visits: Timing, frequency, and determinants of effective antenatal care in Bangladesh - BDHS 2007 to 2017-18
Source: PLoS One. 2025 May 2;20(5):e0321686. doi: 10.1371/journal.pone.0321686 (PMC12047838; doi:10.1371/journal.pone.0321686)
Supplement: S9 Table — (DOCX) [file pone.0321686.s009.docx]

S9 Table: GVIF for binary logistic regression model adjusted for sociodemographic factors number of ANC visits (low (<8) ANC visits) as outcome.

|  | **BDHS 2007** | | | | **BDHS 2017-18** | | | |
| --- | --- | --- | --- | --- | --- | --- | --- | --- |
| **Characteristic** | **GVIF** | **Df** | **Adjusted GVIF** | **Squared Adjusted GVIF** | **GVIF** | **Df** | **Adjusted GVIF** | **Squared Adjusted GVIF** |
| **Area of residence** | 1.41 | 1 | 1.19 | 1.42 | 1.52 | 1 | 1.23 | 1.51 |
| **Wealth index** | 3.03 | 4 | 1.15 | 1.32 | 3.16 | 4 | 1.15 | 1.32 |
| **Region** | 2.06 | 5 | 1.07 | 1.14 | 1.98 | 7 | 1.05 | 1.10 |
| **Women's age** | 2.97 | 1 | 1.72 | 2.96 | 1.97 | 1 | 1.41 | 1.99 |
| **Women’s education level** | 3.87 | 3 | 1.25 | 1.56 | 2.64 | 3 | 1.18 | 1.39 |
| **Women’s employment status** | 1.25 | 1 | 1.12 | 1.25 | 1.34 | 1 | 1.16 | 1.35 |
| **Partner’s education level** | 3.15 | 3 | 1.21 | 1.46 | 2.53 | 3 | 1.16 | 1.35 |
| **Media exposure** | 1.50 | 1 | 1.22 | 1.49 | 1.34 | 1 | 1.16 | 1.35 |
| **Birth order** | 2.98 | 2 | 1.31 | 1.72 | 2.05 | 2 | 1.20 | 1.44 |
| **Distance to health facility** |  |  |  |  | 1.15 | 1 | 1.07 | 1.14 |
| **Owning mobile phone** |  |  |  |  | 1.40 | 1 | 1.18 | 1.39 |
