# Supplementary material for: More than just visits: Timing, frequency, and determinants of effective antenatal care in Bangladesh - BDHS 2007 to 2017-18
Source: PLoS One. 2025 May 2;20(5):e0321686. doi: 10.1371/journal.pone.0321686 (PMC12047838; doi:10.1371/journal.pone.0321686)
Supplement: S1 Text — (DOCX) [file pone.0321686.s011.docx]

**S1 Text: Outcome variables and covariates**

In this study, we investigated two outcome variables with several covariates to assess ANC visits. The outcome variables included are the number of ANC visits and the timing of the first ANC visit. For the number of ANC visits, we categorized the visits into "Less than four" and "Four or more". We also performed the analyses by categorizing the visits into “Less than eight" and "Eight or more" according to the latest WHO recommendations. According to WHO, late antenatal care attendance was defined as attendance after 12 weeks of pregnancy, and based on that, we categorized the time of the first ANC visit into “Late" and "Not late".

Covariates included area of residence (urban or rural), wealth index (poorest, poorer, middle, richer, richest), partner's education level (no education, primary, secondary, higher), respondent's education level (no education, primary, secondary, higher), respondent's working status (working or Not working), media exposure (yes or no ), distance from health facility (not a big problem or big problem), birth order (1, 2-3, 4+), division/region (Barishal, Chattogram, Dhaka, Khulna, Mymensingh, Rajshahi, Rangpur, and Sylhet), women's age at birth (continuous), and owning a mobile phone (yes or no). There is a difference in divisional structure between the 2007 and 20l.17–18 survey rounds, which reflects administrative boundary changes implemented by the Bangladesh Government. In 2007, the survey captured data from six divisions: Barishal, Chattogram, Dhaka, Khulna, Rajshahi, and Sylhet. However, in 2017–18, two additional divisions were included: Rangpur, Mymensingh. Besides the media exposure variable was created based on the exposure of newspaper, radio or television. Those who are said ‘yes’ to any one of these media use, are considered exposed (‘Yes’).
