# Supplementary material for: More than just visits: Timing, frequency, and determinants of effective antenatal care in Bangladesh - BDHS 2007 to 2017-18
Source: PLoS One. 2025 May 2;20(5):e0321686. doi: 10.1371/journal.pone.0321686 (PMC12047838; doi:10.1371/journal.pone.0321686)
Supplement: Attachment — Submitted filename: reviewers comments.pdf [file pone.0321686.s012.pdf]

Dear Editors and Authors,

Thank you for giving me the opportunity to review the manuscript, titled “More Than Just Visits: Timing, Frequency, and Determinants of Antenatal Care in Bangladesh - BDHS 2007 to 2017−18”.

The authors of the study chose a very important topic related to maternal health in Bangladesh. This study examined the factors contributing to delayed ANC and <4 ANC visits, and the association between delayed ANC and <4 ANC. The authors used data from Bangladesh DHS-2007 and DHS2017−18 from women of reproductive age. Geospatial mapping and logistic regression were used to study special pattern and to identify factors associated with delayed ANC and <4 ANC visits. The authors found that late ANC was associated with a significant increase in the odds of <4 ANC visits both in 2007 and in 2017−18.

My assessment of the manuscript is that it is well written. The findings from this study have the potential to impact health interventions and policy to improve initiation of timely ANC visit and use of ANC services in Bangladesh and developing countries. The authors need to address the following issues before the manuscript can be considered for publication.

Throughout the manuscript, the authors should be specified with <4 ANC vs. ≥4 ANC, and should not use the term “adequate or adequacy or inadequacy” because currently WHO recommends ≥8 ANC visits and I don’t think 4-7 ANC visits can be considered adequate based on the revised WHO’s guide.

Abstract

- The purpose of the sentence “… while also evaluating the impact of late ANC initiation on the overall inadequacy of ANC visits.” is not clear, because this study did not examine the impact of late ANC on inadequacy of ANC; but rather examined their associations. This needs to be revised.
- The authors should define the two outcomes of “late ANC, and “<4 ANC visits” in the abstract
- The authors should clarify that they studied the association between the two outcomes, using logistic regression, and provided the ORs of 4.60 for 2007, and 4.68 for 2017-2018, with 95%CIs.

Theoretical framework

- The authors state in lines 101-103 that their analysis explored “accessibility, acceptability, affordability, and awareness of ANC visits” and the data constraints limited the exploration of the dimension of availability”. I’m not sure if this is correct to say all these dimensions. The authors need to state the aspects they actually examined in this study. For example, they did not examine the affordability dimension.

Results

- In Table 1, the authors need to add “total number” for late ANC, and for <4 ANC. The presentation for descriptive statistics is normally column %, and this should be here as well. Currently the percentage for the respective variable becomes 100% only for total sample in 2007 and in 2017-18. This should be the case for the total number for late ANC and for <4 ANC.
- In Table 2, the authors need to add “total number” for late ANC and for <4 ANC. They should also use the term late ANC and <4 ANC, instead of timing of ANC and number of ANC, because they did not use them as continuous variables, but rather binary variables. Also results of 2007 should be presented first in the column, then results from 2017-2018 (the same issue is there in Table 3).
