# Supplementary material for: More than just visits: Timing, frequency, and determinants of effective antenatal care in Bangladesh - BDHS 2007 to 2017-18
Source: PLoS One. 2025 May 2;20(5):e0321686. doi: 10.1371/journal.pone.0321686 (PMC12047838; doi:10.1371/journal.pone.0321686)
Supplement: Attachment — Submitted filename: recommendation.docx [file pone.0321686.s013.docx]

Title: More than just visits: Timing, frequency, and determinants of effective antenatal care in Bangladesh- BDHS 2007 to 2017- 18

Thank you for the opportunity to review your manuscript on a very important topic globally. It was a pleasure to review your manuscript. Please see my feedback below:

Abstract

Methods:

It will be better if you integrate the data in the method section.

Results:

However, age has various effects on ANC, and you mention it in your study, but why wasn’t it considered in the results.

Discussion:

Although there are good arguments about the finding, it is suggested to compare each key finding with other studies and evidence globally or at least other LMICs.

What will be the author’s argument regarding women’s rights to access healthcare during pregnancy and childbirth to reduce the risk of complications, as you mention that women with late ANC and low ANC have more complications during their pregnancy and the probability of MMR?

Why can most women not access healthcare or ANC when women are more educated or the percentage of employed women are increased over the decade? Adding these arguments will enhance the discussion section. (Line 161- 163).

Suggest strengthening the discussion of risk factors for late and low ANC with additional literature.

Why aren’t risk factors of maternal age discussed in the result and discussion section? (line 125 women’s age at birth)

Can you check the reference number 23 ?
