# Supplementary material for: More than just visits: Timing, frequency, and determinants of effective antenatal care in Bangladesh - BDHS 2007 to 2017-18
Source: PLoS One. 2025 May 2;20(5):e0321686. doi: 10.1371/journal.pone.0321686 (PMC12047838; doi:10.1371/journal.pone.0321686)
Supplement: Attachment — Submitted filename: Response to Reviewers.docx [file pone.0321686.s014.docx]

28 February 2024

Muhammad Haroon Stanikzai

Academic Editor

PLOS ONE

RE: PLOS ONE Decision: Revision required [PONE-D-24-52692] - [EMID:989dff884a36553c]

Dear Editor,

Thank you for proving us the opportunity to revise the paper titled “More Than Just Visits: Timing, Frequency, and Determinants of Effective Antenatal Care in Bangladesh - BDHS 2007 to 2017–18” for publication in *PLOS ONE*.

We have addressed the reviewers’ comments in our responses and incorporated related changes into the original manuscript through a revision. For convenience, the reviewers’ comments are highlighted in blue, and our responses are in black font.

The primary changes made in the revised version of the manuscript include:

1. Revised methodology section including variable construction, data description and classification tree method description.
2. Revised results section by updating table format included p-value alongside effect size as requested by the reviewer.
3. Revised discussion section including current literature/references to make comparison of our findings with research conducted in other LMICs and adding policy relevance of our findings.

In addition to the above, all authors have read the revised manuscript. Some minor changes throughout the revised version of the manuscript are made, mostly to improve clarity of expressions and correct grammatical errors.

We would like to thank you and reviewers for all the valuable feedback and suggestions.

Sincere regards,

The authors.

**Title:** More Than Just Visits: Timing, Frequency, and Determinants of Effective Antenatal Care in Bangladesh - BDHS 2007 to 2017–18

Manuscript ID: **PONE-D-24-52692**

# Journal Requirements:

1. Please ensure that your manuscript meets PLOS ONE's style requirements, including those for file naming. The PLOS ONE style templates can be found at <https://journals.plos.org/plosone/s/file?id=wjVg/PLOSOne_formatting_sample_main_body.pdf> and https://journals.plos.org/plosone/s/file?id=ba62/PLOSOne_formatting_sample_title_authors_affiliations.pdf

**Authors’ response**

Thank you for raising the point. We have revised the file names and ensured that our manuscript meets PLOS ONE's style requirements.

2. Please provide additional details regarding participant consent. In the ethics statement in the Methods and online submission information, please ensure that you have specified (1) whether consent was informed and (2) what type you obtained (for instance, written or verbal, and if verbal, how it was documented and witnessed). If your study included minors, state whether you obtained consent from parents or guardians. If the need for consent was waived by the ethics committee, please include this information.

If you are reporting a retrospective study of medical records or archived samples, please ensure that you have discussed whether all data were fully anonymized before you accessed them and/or whether the IRB or ethics committee waived the requirement for informed consent. If patients provided informed written consent to have data from their medical records used in research, please include this information

Once you have amended this/these statement(s) in the Methods section of the manuscript, please add the same text to the “Ethics Statement” field of the submission form (via “Edit Submission”).

For additional information about PLOS ONE ethical requirements for human subjects research, please refer to http://journals.plos.org/plosone/s/submission-guidelines#loc-human-subjects-research.decision??

**Authors’ response**

Thanks for your interest on this important issue. This work does not include any studies with human participants conducted by the authors. Therefore, no ethical approval was needed for our study as we are using secondary data sets provided by the Demographic Health Survey (DHS). The survey protocol for Bangladesh DHS (BDHS) datasets utilized in this study received approval by institutional review boards (IRBs) at ICF and the Bangladesh Medical Research Council (BMRC). Written informed consent was obtained from each participant before conducting the interviews. Participants who did not provide consent were excluded from the survey. Participants were made aware that their participation was entirely voluntary. They were assured that their responses would be kept confidential and anonymous. Additionally, they were informed of their right to skip any questions they preferred not to answer and could withdraw from the interview at any point. All data were fully anonymized before the authors were given access. We have updated the Ethical consideration statement accordingly (page 7 of the revised manuscript).

“**Ethical Considerations**

*This work does not include any studies with human participants conducted by the authors. Therefore, no ethical approval was needed for our study as we are using secondary data sets provided by the Demographic Health Survey (DHS).* The survey protocol for the Bangladesh DHS (BDHS) datasets utilized in this study received *approval by institutional review boards (IRBs) at ICF and the Bangladesh Medical Research Council (BMRC). Written informed consent was obtained from each participant before conducting the interviews. Participants who did not provide consent were excluded from the survey.* Participants were made aware that their participation was entirely voluntary. They were assured that their responses would be kept confidential and anonymous. Additionally, they were informed of their right to skip any questions they preferred not to answer and could withdraw from the interview at any point.  *All data were fully anonymized before the authors were given access.* The datasets are available on request on the Demographic and Health Survey (DHS) website free of cost for research purposes *(*[*https://dhsprogram.com/*](https://dhsprogram.com/)*).*”

3. Please note that your Data Availability Statement is currently missing the repository name. If your manuscript is accepted for publication, you will be asked to provide these details on a very short timeline. We therefore suggest that you provide this information now, though we will not hold up the peer review process if you are unable.

**Authors’ response**

Thank you. We have updated it form the “Data Availability” field of the submission form (via “Edit Submission”).

“*The secondary data sets analyzed during the current study are freely available upon request from the DHS website at* [*https://dhsprogram.com/data/available-datasets*](https://dhsprogram.com/data/available-datasets)*.*”

4. Please include your full ethics statement in the ‘Methods’ section of your manuscript file. In your statement, please include the full name of the IRB or ethics committee who approved or waived your study, as well as whether or not you obtained informed written or verbal consent. If consent was waived for your study, please include this information in your statement as well.

**Authors’ response**

Thank you for raising this point. Ethical approval was not needed for our study since we are using secondary datasets form Bangladesh Demographic and Health Survey (BDHS). “Secondary data vary in terms of the amount of identifying information in it. If the data has no identifying information or is completely devoid of such information or is appropriately coded so that the researcher does not have access to the codes, then it does not require a full review by the ethical board.” (Tripathy, 2013). DHS datasets are popular all over the worlds and there is no identifying information in it, thus it’s not required for us to obtain any additional ethical approval form any IRBs. Besides there are a many existing studies that used this BDHS secondary data sets and published their papers PLOS ONE without further ethical approval for secondary data (Akter et al., 2024; Afiaz et al., 2020; Mansur et al., 2021; Bhowmik et al., 2021).

**References:**

Akter, S., Hosen, M. S., Khan, M. S., & Pal, B. (2024). Assessing the pattern of key factors on women’s empowerment in Bangladesh: Evidence from Bangladesh Demographic and Health Survey, 2007 to 2017–18. *Plos one*, *19*(3), e0301501.

Afiaz, A., Biswas, R. K., Shamma, R., & Ananna, N. (2020). Intimate partner violence (IPV) with miscarriages, stillbirths and abortions: Identifying vulnerable households for women in Bangladesh. *PLoS One*, *15*(7), e0236670.

Mansur, M., Afiaz, A., & Hossain, M. S. (2021). Sociodemographic risk factors of under-five stunting in Bangladesh: Assessing the role of interactions using a machine learning method. Plos one, 16(8), e0256729.

Bhowmik, J., Biswas, R. K., Williams, J., & Dey, S. R. (2024). Women's decision‐making power can influence modern contraceptive use: Evidence from Bangladesh. The International Journal of Health Planning and Management, 39(5), 1503-1515.

Tripathy, J. P. (2013). Secondary data analysis: Ethical issues and challenges. Iranian journal of public health, 42(12), 1478.

5. Please ensure that you refer to Figure 1 and 2 in your text as, if accepted, production will need this reference to link the reader to the figure.

**Authors’ response**

Thank you for the point. We have referred Figure 1 and Figure 2 in the text as follows (page 5 and page 16 of the revised manuscript),

*“While our analysis explores aspects such as accessibility, acceptability, affordability, and awareness of ANC visits, data constraints limit the exploration of the dimension of availability (****Fig 1****).”*

*“The map presented in* ***Fig 2*** *illustrates the regional distribution of late and low (< 4) ANC visits in Bangladesh.”*

6. We note that Figure 2 in your submission contain map/satellite images which may be copyrighted. All PLOS content is published under the Creative Commons Attribution License (CC BY 4.0), which means that the manuscript, images, and Supporting Information files will be freely available online, and any third party is permitted to access, download, copy, distribute, and use these materials in any way, even commercially, with proper attribution. For these reasons, we cannot publish previously copyrighted maps or satellite images created using proprietary data, such as Google software (Google Maps, Street View, and Earth). For more information, see our copyright guidelines: http://journals.plos.org/plosone/s/licenses-and-copyright.

We require you to either (a) present written permission from the copyright holder to publish these figures specifically under the CC BY 4.0 license, or (b) remove the figures from your submission:

a. You may seek permission from the original copyright holder of Figure 2 to publish the content specifically under the CC BY 4.0 license.

We recommend that you contact the original copyright holder with the Content Permission Form (http://journals.plos.org/plosone/s/file?id=7c09/content-permission-form.pdf) and the following text:

“I request permission for the open-access journal PLOS ONE to publish XXX under the Creative Commons Attribution License (CCAL) CC BY 4.0 (http://creativecommons.org/licenses/by/4.0/). Please be aware that this license allows unrestricted use and distribution, even commercially, by third parties. Please reply and provide explicit written permission to publish XXX under a CC BY license and complete the attached form.”

Please upload the completed Content Permission Form or other proof of granted permissions as an "Other" file with your submission.

In the figure caption of the copyrighted figure, please include the following text: “Reprinted from [ref] under a CC BY license, with permission from [name of publisher], original copyright [original copyright year].”

b. If you are unable to obtain permission from the original copyright holder to publish these figures under the CC BY 4.0 license or if the copyright holder’s requirements are incompatible with the CC BY 4.0 license, please either i) remove the figure or ii) supply a replacement figure that complies with the CC BY 4.0 license. Please check copyright information on all replacement figures and update the figure caption with source information. If applicable, please specify in the figure caption text when a figure is similar but not identical to the original image and is therefore for illustrative purposes only.

The following resources for replacing copyrighted map figures may be helpful:

USGS National Map Viewer (public domain): http://viewer.nationalmap.gov/viewer/

The Gateway to Astronaut Photography of Earth (public domain): http://eol.jsc.nasa.gov/sseop/clickmap/

Maps at the CIA (public domain): https://www.cia.gov/library/publications/the-world-factbook/index.html and https://www.cia.gov/library/publications/cia-maps-publications/index.html

NASA Earth Observatory (public domain): http://earthobservatory.nasa.gov/

Landsat: http://landsat.visibleearth.nasa.gov/

USGS EROS (Earth Resources Observatory and Science (EROS) Center) (public domain): http://eros.usgs.gov/#

Natural Earth (public domain): http://www.naturalearthdata.com/

**Authors’ response**

Apologies for any confusion. Figure 2 of our manuscript contain spatial mappings. This map is not copyrighted. This is original image created by us using the statistical software R. The R packages and shapefile used in this manuscript are listed on **page 7**; in the last paragraph of the Statistical analyses section. Besides, we did not use any R packages that source their maps from Google Maps. Instead, we utilized the administrative boundary shapefile of South Asian countries from an openly licensed database, the geoBoundaries Global Administrative Database (Runfola et al., 2020), which we have cited in the last paragraph of the Statistical analyses section.

**Reference:**

Runfola D, Anderson A, Baier H, Crittenden M, Dowker E, Fuhrig S, Goodman S, Grimsley G, Layko R, Melville G, Mulder M. geoBoundaries: A global database of political administrative boundaries. PloS one. 2020 Apr 24;15(4):e0231866.

7. We note you have included a table to which you do not refer in the text of your manuscript. Please ensure that you refer to Table 2 in your text; if accepted, production will need this reference to link the reader to the Table.

**Authors’ response**

Thank you for noticing this. We have updated our manuscript and referred Table 2 and, in the text, as follows (page 11 of revised manuscript),

*“The results of binary logistic regression model adjusted for sociodemographic factors with timing of first ANC visit and number of ANC visits (low (< 4) ANC visits) as outcome is shown in* ***Table 2****.”*

8. Please include captions for your Supporting Information files at the end of your manuscript, and update any in-text citations to match accordingly. Please see our Supporting Information guidelines for more information: http://journals.plos.org/plosone/s/supporting-information.

**Authors’ response**

Thank you for raising the point. We have updated the manuscript accordingly and the following section has been added at the end of our manuscript (page 25 of the revised manuscript),

*“****Supporting information***

***S1 Table. The frequency distribution of late ANC and low (< 8) ANC visits by sociodemographic factors.***

***S2 Table. The frequency distribution of low (<8) ANC visits by timing of first ANC visits.***

***S3 Table. Binary logistic regression model adjusted for sociodemographic factors and timing of first ANC visit with number of ANC visits (low (<4) ANC visits) as outcome.***

***S4 Table. Binary logistic regression model adjusted for sociodemographic factors with timing of first ANC visit and number of ANC visits (low (<8) ANC visits) as outcome.***

***S5 Table. Binary logistic regression model adjusted for sociodemographic factors and timing of first ANC visit with number of ANC visits (low (<8) ANC visits) as outcome.***

***S6 Table.*** ***GVIF for binary logistic regression model adjusted for sociodemographic factors with timing of first ANC visit as outcome.***

***S7 Table. GVIF for binary logistic regression model adjusted for sociodemographic factors with number of ANC visits (low (<4) ANC visits) as outcome.***

***S8 Table. GVIF for binary logistic regression model adjusted for sociodemographic factors and timing of first ANC visit with number of ANC visits (low (<4) ANC visits) as outcome***

***S9 Table. GVIF for binary logistic regression model adjusted for sociodemographic factors number of ANC visits (low (<8) ANC visits) as outcome.***

***S10 Table. GVIF for binary logistic regression model adjusted for sociodemographic factors and timing of first ANC visit with number of ANC visits (low (<8) ANC visits) as outcome***

***S1 Text. Outcome variables and covariates****”*

# Additional Editor Comments:

- Please revise categories for media exposure (yes vs no).

**Authors’ response**

Thank you for raising the point. We have revised the categories for media exposure (yes vs no). We have put the details in supplementary S1 text,

*“Besides the media exposure variable was created based on the exposure of newspaper, radio or television. Those who are said ‘yes’ to any one of these media use, are considered exposed (‘Yes’).”*

-Please revise categories for mobile ownership (yes vs no).

**Authors’ response**

Thank you for raising the point. We have revised the categories for mobile ownership (yes vs no). We have made the changes in methods and tables (Table 1, Table 2 and supplementary tables),

-It would be desirable for the authors to mention in introduction why it is important for the population of women studied in Bangladesh to analyze access to mobile phones.

**Authors’ response**

Thank you for the suggestion. We have revised the introduction section of our manuscript and added why we believe it is important for the population of women studied in Bangladesh to analyze access to mobile phones. Following text has been added in page 4 of the revised manuscript,

“*Additionally owning mobile phones also significantly plays a critical role in improving maternal health outcomes in low- and middle-income countries (LMICs) like Bangladesh. Studies have found that mobile phones help better access to health information, ANC visits, emergency health communication and improving overall awareness (12,13).*”

-Line 264-266: Suggest to add citation: https://journals.plos.org/plosone/article?id=10.1371/journal.pone.0309300.

**Authors’ response**

Thank you for the suggestion. We have added this citation in the mentioned line. Following citation has been added in the revised manuscript,

*“**These results are particularly pertinent to LMICs such as Bangladesh, where traditional societal norms and cultural beliefs exert a substantial influence on healthcare-seeking behaviors (33,34)”*

*“34. Stanikzai MH, Tawfiq E, Suwanbamrung C, Wasiq AW, Wongrith P. Predictors of antenatal care services utilization by pregnant women in Afghanistan: Evidence from the Afghanistan Health Survey 2018. PLoS One. 2024 Oct 1;19(10):e0309300.”*

# Reviewer #1

**Reviewer’s comment**

This Major Concerns:

1. Methodological Issues:

The authors need to better justify their choice of cutoff points for "late" ANC initiation. While they follow national guidelines, a discussion of alternative definitions used in the literature would strengthen the paper. The potential for recall bias in self-reported data should be more thoroughly addressed in the limitations section.

**Authors’ response**

Thank you for pointing out these concerns.

Regarding the definition of “Late” ANC initiation, we have followed the WHO FANC model and 2016 guideline for the **timing of first** ANC visit (WHO consider ANC to be late if the first ANC visit takes place after 12 weeks). However, according to Bangladeshi National Guidelines, an ANC visit is considered late if it takes place after the first 16 weeks of pregnancy.

On the other hand, WHO updated its minimum recommended number of antenatal care (ANC) visits from four (4) to eight (8) visits in 2016 as part of its revised guidelines, whereas Bangladesh National guidelines still recommend four visits. Yet, both versions of the WHO FANC models suggested the same definition for Late ANC (if first visit is after 12 weeks).

There to be consistent with the literature and better generalizability of the results on a global standard across LMICs, we followed the WHO’s recommendations to define Late ANC (>12 weeks) and Low ANC (>4 and >8 visits). We focused primarily on >4 visits to define Low ANC, as this coincides with Bangladesh’s national guidelines and the fact that the data comes from the 2007 and 2017-18 BDHS. Furthermore, we have also conducted a full analysis with considering the >8 visits threshold for Low ANC based on WHO’s latest recommendation and provided the full results in the supplemental document (**S1 Table, S2 Table, S4 Table, S5 Table, S9 Table** and **S10 Table.**). Both definitions provided similar results with the >8 visit threshold providing larger effect sizes, as expected.

We have revised the outcome variable section by defining which guideline we followed for constructing the variables. Following text has been added in page 5-6 of the revised manuscript,

“*While Bangladesh national guidelines define late ANC initiation as the first visit occurring after 16 weeks of gestation, this study adopted WHO’s stricter >12-week threshold to align with global standards and enhance cross-LMIC comparability. For ANC frequency (i.e. Low ANC), we analyzed both WHO’s updated 8-visit benchmark and Bangladesh’s 4-visit guideline, prioritizing the latter in primary analyses to reflect local policy and the 2007 and 2017-18 BDHS datasets. Supplemental use of the WHO 8-visit threshold produced directionally consistent results but amplified the effect sizes (detailed in* ***S1 Table, S2 Table, S4 Table,*** *and* ***S5 Table****).*”

2. Analytical Concerns:

The classification tree analysis, while innovative, requires more detailed explanation of the methodology and interpretation criteria. The spatial analysis would benefit from statistical tests of spatial autocorrelation to support the observed patterns.

**Authors’ response**

We thank the reviewer for these suggestions. We have added the methodology and interpretation criteria for classification tree in the methodology section. Following text has been added in page 7 of the revised manuscript,

“*We utilized classification tree analysis to investigate complex interactions between predictors and nonlinear relationships between predictor variables and the outcome measures (23). It partitions data using hypothesis testing at each node. The null hypothesis is* $H_{0j}:D\left( \boldsymbol{Y}|X_{j} \right)=D\left( \boldsymbol{Y} \right)$*, which assesses whether the response variable* $\boldsymbol{Y}$ *is independent of each covariate* $X_{j};$ *j = 1, 2, …, m. The test statistics is defined as:*

$$T_{j}\left( L_{n},w \right)=\text{vec}\left( \sum_{i=1}^{n} w_{i}g_{j}\left( X_{ji} \right)h\left( Y_{i},{(Y}_{1},Y_{2},\ldots,Y_{n} \right))' \right),$$

*where* $g_{j}\left( X_{ji} \right)$ *is a transformation of the predictor,* $h\left( Y_{i},{(Y}_{1},Y_{2},\ldots,Y_{n} \right))'$ *represents the influence function of the response, and* $w_{i}$ *denotes case weights. The test statistic is standardized using its conditional expectation (*$\mu_{j}$*) and covariance (*$\Sigma_{j}$*) under the null hypothesis. If* $H_{0j}$ *is rejected, the algorithm selects the covariate with the strongest association (smallest p-value). In the next step it determines the optimal split by maximizing a discrepancy measure:*

$$A^{*}=arg \max_{A} c\left( T_{A},\mu_{A},\Sigma_{A} \right)$$

*The recursion halts when no significant association is found at a predefined significance level (*$\alpha$*).*

*The results of the classification tree analysis provide useful insights. The tree structure visually interprets the importance of covariates in splitting data. The significance of splits is determined by p-values, with corrections like Bonferroni adjustments applied to handle multiple comparisons. Terminal nodes provide a summary of the response variable within the distinct partitions.*”

Please note that a full spatial analysis is out of the scope of this paper. We provided the spatial maps in the form of weighted prevalence mapping to understand the hotspots for better contextualization of the results. However, we note that this is a limitation and so it has been acknowledged in discussion. Following text has been added in page 20 of the revised manuscript,

“*Finally, future research can consider applying models such as Spatial Lag Models to study the spatial autocorrelations of ANC services in Bangladesh.*”

3. Results Presentation:

Some figures (particularly Figure 2) need clearer labeling and legend explanations. The tables would benefit from consistent formatting and clearer presentation of confidence intervals.

**Authors’ response**

Thank you for pointing this out. We have revised Figure 2, and it now includes clearer labeling to enhance its readability. Please refer to the revised map below:

Additionally, we have updated the presentation of the tables to ensure consistent formatting and provide a clearer display of confidence intervals. Please refer to Table 2 and Table 3.

4. Technical Corrections:

Throughout the manuscript, several acronyms are not defined at first use. There is inconsistent use of decimal places in reported statistics. Some references need updating to more recent sources to reflect current understanding of the field.

**Authors’ response**

Thank you for pointing this out. We have checked and updated acronyms throughout the manuscript. We have reported up to 1 decimal places for reported prevalence, 2 decimal places for AOR and CIs of AOR and three decimal places for p-value, following the extant literature. We have further checked for consistency throughout the manuscript.

We have also provided the references from more recent (mostly in the discussion section). Please refer to the revised reference section in page 21 of the revised manuscript.

Specific Recommendations:

5. Introduction:

The rationale for comparing 2007 and 2017-18 specifically needs strengthening. Additional context about global ANC recommendations and how Bangladesh's guidelines differ would enhance the reader's understanding of the study's framework.

**Authors’ response**

To assess the evolution of antenatal care (ANC) service structure in Bangladesh over a decade, we specifically selected data from the 2007 and 2017–18 survey waves. This choice was driven by the need to capture long-term trends in maternal healthcare provision, reflecting policy shifts, healthcare infrastructure improvements, and changes in service utilization patterns. By comparing these two time points, we aimed to evaluate the extent to which ANC service delivery has transformed in response to national health initiatives, demographic transitions, and broader socioeconomic changes. We added the following in page 4 of the revised manuscript,

“*We specifically wanted to assess the change in ANC service structure in Bangladesh across one decade and thus we chose to use 2007 and 2017-18 survey results.*”

Besides, we have revised the outcome variable section by defining which guideline we followed for constructing the variables. As mentioned, we have also updated the discussion to highlight that the Bangladesh national guidelines should be revisited to align with global standards, facilitating cross-country comparisons and the adoption of global strategies in page 17 of the revised manuscript,

“*Furthermore, Bangladesh national guidelines need to revisit the recommended number and timing of ANC visits to align with WHO standards. This would enhance the understanding, help in cross-country comparisons and adapting global strategies to improve maternal health outcomes.*”

6. Methods:

The authors should provide more detail about the handling of missing data and clarify the criteria for inclusion/exclusion of variables in the regression models. The choice of classification tree parameters requires additional explanation to ensure reproducibility.

**Authors’ response**

Given we utilized large-scale survey data, missing cases were addressed through listwise deletion, assuming that the data were missing at random to ensure unbiased estimates, which is consistent with literature of nationally representative surveys.

References:

1. Vollmer S, Harttgen K, Kupka R et al. Levels and trends of childhood undernutrition by wealth and education according to a composite index of anthropometric failure: evidence from 146 demographic and health surveys from 39 countries. BMJ Glob Health 2017;2(2):e000206.
2. Biswas, R.K., Arusha, A.R., Ananna, N., Kabir, E. and Bhowmik, J., 2023. Carer involvement with children and child‐friendly book ownership in Bangladesh. Children & Society, 37(2), pp.326-342.
3. Bhowmik, J., Apputhurai, P., Williams, J. and Biswas, R.K., 2024. Investigating the role of women's education status and empowerment on accessing skilled birth attendance in Bangladesh: A structural equation modelling approach. Midwifery, 138, p.104140.
4. Bhowmik, J., Biswas, R.K., Williams, J. and Dey, S.R., 2024. Women's decision‐making power can influence modern contraceptive use: Evidence from Bangladesh. The International Journal of Health Planning and Management, 39(5), pp.1503-1515.
5. Biswas, R.K., Farzana, M., Bhar, S. and Bhowmik, J., 2023. Contraceptive use in South and South-East Asian region: assessment of sustainable development goal 3.7 through indicator 3.7. 1. Journal of Public Health, 45(2), pp.450-461.

As mentioned above, we have added the methodology and interpretation criteria for classification tree in the methodology section. Please refer to the following text in page 7 of the revised manuscript,

“*We utilized classification tree analysis to investigate complex interactions between predictors and nonlinear relationships between predictor variables and the outcome measures (25). It partitions data using hypothesis testing at each node. The null hypothesis is* $H_{0j}:D\left( \boldsymbol{Y}|X_{j} \right)=D\left( \boldsymbol{Y} \right)$*, which assesses whether the response variable* $\boldsymbol{Y}$ *is independent of each covariate* $X_{j};$ *j = 1, 2, …, m. The test statistics is defined as:*

$$T_{j}\left( L_{n},w \right)=\text{vec}\left( \sum_{i=1}^{n} w_{i}g_{j}\left( X_{ji} \right)h\left( Y_{i},{(Y}_{1},Y_{2},\ldots,Y_{n} \right))' \right),$$

*where* $g_{j}\left( X_{ji} \right)$ *is a transformation of the predictor,* $h\left( Y_{i},{(Y}_{1},Y_{2},\ldots,Y_{n} \right))'$ *represents the influence function of the response, and* $w_{i}$ *denotes case weights. The test statistic is standardized using its conditional expectation (*$\mu_{j}$*) and covariance (*$\Sigma_{j}$*) under the null hypothesis. If* $H_{0j}$ *is rejected, the algorithm selects the covariate with the strongest association (smallest p-value). In the next step it determines the optimal split by maximizing a discrepancy measure:*

$$A^{*}=arg \max_{A} c\left( T_{A},\mu_{A},\Sigma_{A} \right)$$

*The recursion halts when no significant association is found at a predefined significance level (*$\alpha$*).*

*The results of the classification tree analysis provide useful insights. The tree structure visually interprets the importance of covariates in splitting data. The significance of splits is determined by p-values, with corrections like Bonferroni adjustments applied to handle multiple comparisons. Terminal nodes provide a summary of the response variable within the distinct partitions.*”

7. Results:
   Effect sizes should be included alongside p-values consistently throughout the results section. The authors should expand their analysis to include more detailed subgroup analyses for urban/rural differences. Sensitivity analyses for different definitions of "late" ANC would strengthen the robustness of the findings.

**Authors’ response**

Thanks for your this important suggestion. We have updated our result section by including the p-values alongside the effect sizes (page 11-12 of the revised manuscript). We agree that subgroup analysis would be interesting. However, our aim of the study was to observe changes in one decade across late and insufficient ANC. We believe that such subgroup analysis could be a potential research question for further studies in this topic.

“**Logistic regression models**

The results of binary logistic regression model adjusted for sociodemographic factors with timing of first ANC visit and number of ANC visits (low (< 4) ANC visits) as outcome is shown in **Table 2**. Among urban and rural mothers, in 2007, rural mothers were associated with 26% higher odds (AOR: 1.26 [95% CI: 1.03, 1.53]; *p-value = 0.023*) of having a late ANC visit compared to urban mothers. In terms of the number of ANC visits, rural mothers were associated with 30% (AOR: 1.30 [1.08, 1.55]; *p-value = 0.005*) and 71% (AOR: 1.71 [1.38, 2.11]; *p-value < 0.001*) higher odds of having low ANC visits, respectively, in 2017–18 and 2007, compared to urban mothers. For each one-year difference in women's age, older women were associated with 2% lower (AOR: 0.98 [95% CI: 0.96, 1.00*]; p-value = 0.036*) and 3% lower odds (AOR: 0.97 [95% CI: 0.95, 0.99]; *p-value = 0.003*) of having a late ANC visit in 2017–18 and 2007, respectively. Similarly, the odds of having a low number of ANC visits were found lower by 3% for each additional year of age in both 2017–18 (AOR: 0.97 [95% CI: 0.95, 0.99]; *p-value < 0.001*) and 2007 (AOR: 0.97 [95% CI: 0.95, 0.99]; *p-value = 0.011*). Despite the survey year, the poorest, least educated, and, in 2017–18, no media-exposed women were associated with higher odds of having late and low number of ANC visits compared to the reference categories. Besides, for 2017–18, those for whom distance to health facilities was a problem were associated with 20% higher odds (AOR: 1.20 [1.03, 1.38]; *p-value = 0.016*) of having low ANC visits compared to those who did not have any problem with the distance. In the case of women owning mobile phones, there were, respectively, 16% (AOR: 0.84 [0.72, 0.97]; *p-value = 0.021*) and 23% (AOR: 0.77 [0.66, 0.89]; *p-value < 0.001*) lower odds of having late and low ANC visits compared to women without a mobile phone. Irrespective of the survey year, in the case of the highest birth order, mothers were associated with higher odds of having late and low ANC visits compared to if they were giving birth to a first, second, or third child. Women with higher-educated partners were also associated with lower odds of having late and low ANC coverage compared to those having partners with primary or no education.

In 2017–18, women who reported late ANC initiation were associated with 4.68 times (AOR: 4.68 [95% CI: 4.00, 5.48]; *p-value < 0.001*) the odds of having less than four ANC visits compared to those who were not late after adjusting for the others covariate, which remained unchanged in 2007 (AOR: 4.60 [95% CI: 3.69, 5.73]; *p-value < 0.001*), shown in **Table 3** and detailed in **S3 Table**.”

8. Discussion:

The policy implications of the findings need expansion, particularly regarding practical implementation strategies. The comparison with other low- and middle-income countries should be strengthened to provide broader context. The sustainability of observed improvements requires more thorough examination.

**Authors’ response**

We did not delve too much into the practical implementation as this was a cross-sectional design. Our study also lacks qualitative analysis to provide policy level discussion. We have added the following paragraph in page 19 of the revised manuscript,

“*Although the study is cross-sectional, valuable policy insights can be drawn from the findings. Strengthening community-based interventions, such as targeted health education campaigns and home visits by community health workers, can improve awareness and encourage early ANC initiation. Integrating ANC services within existing maternal and child health programs and ensuring financial and geographic accessibility, particularly for socioeconomically disadvantaged groups, may reduce barriers to care. Recognizing women's right to access healthcare during pregnancy and childbirth is essential to reducing the risk of complications and ensuring equitable maternal health outcomes. Policymakers should consider revising national ANC guidelines to emphasize early initiation and continuity of care at the global standard, ensuring they are effectively disseminated and implemented across all healthcare facilities.*”

# Reviewer #2

**Reviewer’s comment**

1. Throughout the manuscript, the authors should be specified with <4 ANC vs. ≥4 ANC, and should not use the term “adequate or adequacy or inadequacy” because currently WHO recommends ≥8 ANC visits and I don’t think 4-7 ANC visits can be considered adequate based on the revised WHO’s guide.

**Authors’ response**

Thank you for your suggestion. We have revised the manuscript accordingly. The terms have been used in the abstract, introduction, and discussion. While we used 'adequate' and 'inadequate' to indirectly refer to individuals receiving the recommended number of ANC visits, these terms were not used in the results section to avoid confusion with other visit-related findings. For example, an analysis comparing <8 ANC visits to ≥8 ANC visits was included in the supplementary section, and the findings were consistent with those for <4 ANC visits vs. ≥4 ANC visits.

Abstract

2. The purpose of the sentence “… while also evaluating the impact of late ANC initiation on the overall inadequacy of ANC visits.” is not clear, because this study did not examine the impact of late ANC on inadequacy of ANC; but rather examined their associations. This needs to be revised.

**Authors’ response**

Thank you for pointing this. We have revised the mentioned sentence in page 2 of the revised manuscript to improve the clarity as follows:

*“This study examines the factors contributing to* *delayed initiation and a low number of ANC visits, while also evaluating the association between the timing and overall number of ANC visits.”*

3. The authors should define the two outcomes of “late ANC, and “<4 ANC visits” in the abstract.

**Authors’ response**

Thank you for your suggestion. We have revised the abstract by adding the following sentence in page 2 of the revised manuscript,

*“We investigated two binary outcome variables: late ANC, defined as the initiation of ANC visits after 12 weeks of gestation, and low ANC, defined as having less than four ANC visits.”*

4. The authors should clarify that they studied the association between the two outcomes, using logistic regression, and provided the ORs of 4.60 for 2007, and 4.68 for 2017-2018, with 95%CIs.

**Authors’ response**

Thank you for your suggestion. We have revised the abstract by adding the following sentence in page 2 of the revised manuscript,

*“Logistic regression modeling revealed that late ANC was associated with a more than fourfold increase in the odds of having fewer than four ANC visits (AOR: 4.60 [95% CI: 3.69–5.73] in 2007 and AOR: 4.68 [95% CI: 4.00–5.48] in 2017–18).”*

Theoretical framework

5. The authors state in lines 101-103 that their analysis explored “accessibility, acceptability, affordability, and awareness of ANC visits” and the data constraints limited the exploration of the dimension of availability”. I’m not sure if this is correct to say all these dimensions. The authors need to state the aspects they actually examined in this study. For example, they did not examine the affordability dimension.

**Authors’ response**

Thank you for raising this point. We have clarified that we did not have any proxy variable to address the dimension of availability, whereas affordability can be addressed through family financial status and women’s working status. The data source for this study is secondary in nature, therefore we are compelled to work with certain data insufficiency as a result. We have noted this fact in the limitations paragraph at the end of the discussion. Please refer to Figure 1 provided below:

Results

6. In Table 1, the authors need to add “total number” for late ANC, and for <4 ANC. The presentation for descriptive statistics is normally column %, and this should be here as well. Currently the percentage for the respective variable becomes 100% only for total sample in 2007 and in 2017-18. This should be the case for the total number for late ANC and for <4 ANC.

**Authors’ response**

Thank you for your suggestion. We have added the sample size for Late ANC and <4 ANC in the last six rows of Table 1. However, the percentages for the Late ANC and <4 ANC columns at the levels of any specific covariates should not sum to 100%. This is because these percentages represent the row-wise distribution of the occurrence of Late ANC and <4 ANC across covariate levels, rather than a column-wise distribution. The percentages are calculated based on the total number of individuals at each covariate level, which provides a more meaningful and convenient way to interpret the data.

| **Variables** | **BDHS 2017-18** | | | **BDHS 2007** | | |
| --- | --- | --- | --- | --- | --- | --- |
|  | **Total sample,**  **N = 4,588 (%)** | **N (%) of women with Late ANC visits** | **N (%) of women with Low (< 4) ANC**  **visits** | **Total sample,**  **N = 2,949** | **N (%) of women with Late ANC visits** | **N (%) of women with Low (< 4) ANC**  **visits** |
| **Area of residence** |  | | |  | | |
| Urban | 1,266 (27.6) | 634 (50.0) | 479 (37.8) | 783 (26.5) | 379 (48.4) | 387 (49.5) |
| Rural | 3,322 (72.4) | 2,104 (63.3) | 1,761 (53.0) | 2,166 (73.5) | 1,377 (63.6) | 1,553 (71.7) |

Our primary objective is to assess the prevalence of Late ANC and <4 ANC across different covariate categories. The current table format allows us to clearly convey insights such as:

We can able to conclude here that in both 2007 and 2017–18, rural women had higher rates of Late ANC visits (15.2% and 13.3%, respectively) and also reported a greater percentage of <4 ANC visits (22.2% and 15.2%, respectively) compared to women in urban areas.

If we were to adopt the suggested column-wise percentage format, the interpretation would change:

| **Variables** | **BDHS 2017-18** | | | **BDHS 2007** | | |
| --- | --- | --- | --- | --- | --- | --- |
|  | **Total sample,**  **N = 4,588 (%)** | **Late ANC visits N = 2,737 (%)** | **Low (< 4) ANC visits N= 2,240 (%)** | **Total sample,**  **N = 2,949 (%)** | **Late ANC visits N = 1,756 (%)** | **Low (< 4) ANC**  **visits N =** 1,940 **(%)** |
| **Area of residence** |  | | |  | | |
| Urban | 1,266 (27.6) | 634 (23.2) | 479 (21.4) | 783 (26.5) | 379 (21.6) | 387 (20.0) |
| Rural | 3,322 (72.4) | 2,103(76.8) | 1,761 (78.6) | 2,166 (73.5) | 1,377 (78.4) | 1,553 (80.0) |

Now from this version of table we can say, among the women in 2017–18 who started their ANC visits late, 23.2% were from urban areas, while 76.8% were from rural areas. This alternative format does not provide information on the percentage of rural and urban women who experienced Late ANC or <4 ANC visits. Instead, it focuses on the distribution of these outcomes within the subgroup of women who had Late ANC or <4 ANC visits, which is not the focus of our analysis.

Furthermore, several previous research studies have used a similar table format, supporting the appropriateness of our existing representation approach. Some of these studies are listed below for reference.

**References:**

Ahmed, M. S., Islam, M. I., Das, M. C., Khan, A., & Yunus, F. M. (2021). Mapping and situation analysis of basic WASH facilities at households in Bangladesh: evidence from a nationally representative survey. *PLOS One*, *16*(11), e0259635.

Afiaz, A., Biswas, R. K., Shamma, R., & Ananna, N. (2020). Intimate partner violence (IPV) with miscarriages, stillbirths and abortions: identifying vulnerable households for women in Bangladesh. *PLOS One*, *15*(7), e0236670.

Afiaz, A., & Biswas, R. K. (2021). Awareness on menstrual hygiene management in Bangladesh and the possibilities of media interventions: using a nationwide cross-sectional survey. *BMJ open*, *11*(4), e042134.

Mansur, M., Afiaz, A., & Hossain, M. S. (2021). Sociodemographic risk factors of under-five stunting in Bangladesh: Assessing the role of interactions using a machine learning method. *PLOS One*, *16*(8), e0256729.

7. In Table 2, the authors need to add “total number” for late ANC and for <4 ANC. They should also use the term late ANC and <4 ANC, instead of timing of ANC and number of ANC, because they did not use them as continuous variables, but rather binary variables. Also results of 2007 should be presented first in the column, then results from 2017-2018 (the same issue is there in Table 3).

.

**Authors’ response**

Thank you for the suggestion. The “total number” for Late ANC and <4 ANC is already included in Table 1, where the last six rows provide the mentioned information.

We have revised the terms timing of ANC and number of ANC to your suggested terms, Late ANC and <4 ANC, to accurately reflect that these variables are binary rather than continuous.

Furthermore, we have revised all tables, including Table 1, Table 2, Table 3, and all supplementary tables, to present the results of 2007 first, followed by the results of 2017–18, as you suggested.

# Reviewer #3

**Reviewer’s comments**

Methods:

1. It will be better if you integrate the data in the method section.

**Authors’ response**

Thank you for your suggestion. We have revised the data overview section in the methods section, and it now clearly providing the details about the data we have used (page 5 of the revised manuscript),

*“****Data overview***

*The data used in this analysis comes from the Bangladesh Demographic and Health Survey (BDHS).* BDHS is a part of the Demographic Health Survey program conducted in LMICs by USAID, collects accurate, nationally representative data on fertility, family planning, maternal and child health, gender, HIV/AIDS, malaria, and nutrition(22). *We utilized the Bangladesh Demographic and Health Survey (BDHS) data sets from 2007 (n = 3050) and 2017–18 (n = 4544). Both of these two BDHS 2007 and 2017–18 surveys were chosen to allow for a comparative analysis over a decade, capturing potential changes in antenatal care (ANC) utilization patterns and associated sociodemographic factors over time. The sampling frame for both surveys consisted of a list of enumeration areas (EAs) derived from the census. Employing a two-stage stratified cluster sampling approach with probability proportional to size sampling method, the initial stage comprised selecting 361 EAs in 2007 and 675 EAs in 2017-18. Subsequently, a second stage involved employing an equal probability systematic sampling method to select an average of 30 households from each cluster. In the 2017-18 BDHS, data collection occurred in a total of 20,160 households and 10,819 households made up the sample of data for the 2007 BDHS.”*

Results:

2. However, age has various effects on ANC, and you mention it in your study, but why wasn’t it considered in the results.

**Authors’ response**

Thank you for pointing out this issue. We have revised the result section, and it now includes description about significant association with both late and low ANC visits (page 11 of the revised manuscript),

*“**For each one-year difference in women's age, older women were associated with 2% lower (AOR: 0.98 [95% CI: 0.96, 1.00]; p-value = 0.036) and 3% lower odds (AOR: 0.97 [95% CI: 0.95, 0.99]; p-value = 0.003) of having a late ANC visit in 2017–18 and 2007, respectively. Similarly, the odds of having a low number of ANC visits were found lower by 3% for each one year of age in both 2017–18 (AOR: 0.97 [95% CI: 0.95, 0.99]; p-value < 0.001) and 2007 (AOR: 0.97 [95% CI: 0.95, 0.99]; p-value = 0.011).”*

Discussion:

3. Although there are good arguments about the finding, it is suggested to compare each key finding with other studies and evidence globally or at least other LMICs.

**Authors’ response**

Thank you for your suggestion. We initially included the key variable that can be intervened or actionable policy wise. However, now we have revised the discussion section, and it now includes the comparison of each key finding with other studies and evidence globally or at least other LMICs. Please refer to the revised discussion section in page 18-20.

“**Discussion**

Our study explored the association between late initiation of ANC and subsequent low ANC attendance, highlighting the critical importance of early ANC visits and their relationship with achieving the recommended number of ANC visits. We identified sociodemographic factors associated with both *timing and total number* of ANC visits. The findings revealed that, in both 2007 and 2017–18, approximately 60% of women delayed their first ANC visit, with a slight increase observed over the decade. While crude prevalence estimates for low ANC visits decreased by 17%, nearly half of the women (approximately 50%) still did not meet the minimum number of ANC visits recommended by the WHO FANC model and national guidelines in Bangladesh. Importantly, the factors associated with both late and insufficient ANC visits remained largely unchanged over the 10-year period. Our results underscore that, despite governmental efforts to improve maternal healthcare, sociodemographic factors such as education, socioeconomic status, maternal age, and birth order have continued to influence the timing and frequency of ANC visits throughout this period. *Furthermore, Bangladesh national guidelines need to revisit the recommended number and timing of ANC visits to align with WHO standards. This would enhance the understanding, help in cross-country comparisons and adapting global strategies to improve maternal health outcomes.*

Our analysis revealed a strong association between the timing of the first ANC visit and the subsequent number of ANC visits, as evidenced by the regression model results. The classification tree analysis further confirmed that the timing of the first ANC visit was the most significant predictor of *number of ANC visits*, with consistent findings across both the 2007 and 2017–18 surveys. These results are particularly pertinent to LMICs such as Bangladesh, where traditional societal norms and cultural beliefs exert a substantial influence on healthcare-seeking behaviors (33,34). In Bangladeshi rural areas, pregnancy is often regarded as a natural process that does not require early medical intervention unless complications arise, leading to delayed ANC initiation (35). Additionally, decision-making regarding maternal health is frequently influenced by husbands or elder family members (33,35), who may not prioritize early ANC visits due to traditional views or a lack of awareness. Furthermore, rural women, who are the most susceptible to being influenced by traditional norms, also showed higher odds of late and low ANC visits compared to urban mothers, irrespective of the survey year, a trend supported by studies in Bangladesh (36) and Vietnam (37). *Additionally, women facing challenges with distance to healthcare facilities showed higher odds of having a low number of ANC visits. Studies conducted in Burkina Faso (38), and Benin (39) further support to this finding.*

*Despite increases in women’s education and employment, many women in Bangladesh continue to face significant barriers in accessing healthcare and ANC. Issues including cultural norms, poor healthcare facilities, and financial challenges make it difficult for women in accessing healthcare facilities (40).* Women from less affluent households had higher odds of having late initiation and a low number of ANC visits compared to the others, indicating socioeconomic inequalities in ANC coverage in Bangladesh. Financial barriers significantly impact access to maternal healthcare services, as lower-income families may prioritize immediate basic needs over healthcare expenses, leading to delayed or irregular ANC visits (41,42)*. Evidence from studies in Ethiopia (43,44), and Cameroon (17) also supports this conclusion.* While most regions have shown improvement over the decade, regional disparities still exist, highlighting the ongoing need to address regional inequalities in maternal healthcare access in Bangladesh, as evident from recent studies (45). *Regional variations in ANC utilization also evident in other LMICs like Ethiopia (46) and Indonesia (47).* Women with higher education levels had reduced odds of experiencing late initiation and insufficient ANC visits compared to those with lower levels of education which underscored the crucial role of mother’s education in promoting timely and adequate ANC attendance (41,48,49). *This assertion finds additional backing from research conducted in other LMICs including Nigeria (50,51), Ethiopia (15,38,46), and Indonesia (47).*

Our findings underscored the importance of accessing information and healthcare facilities in ensuring timely and adequate ANC visits. Access to information through media and mobile phones enhances awareness and understanding of the importance of early and regular ANC visits, ultimately improving maternal healthcare utilization (41,48), highlighting the importance of media exposure, internet use, and mobile phones on reproductive healthcare services. *A recent study in Bangladesh also highlights the crucial role of internet use, and mobile phone on reproductive health service (52). Maternal age also has been identified as a significant factor associated with both the timing and number of ANC visits. Previous study conducted in Ethiopia (53), Ghana (54), and Gambia (55) also found maternal age associated with timing and number of ANC visit.*

We found that women who gave birth to three or more children previously had increased odds of experiencing late initiation and *<4 ANC visits* compared to those who had less than two children. This phenomenon can be attributed to several factors. Firstly, mothers with multiple children often face increased household responsibilities (56) and time constraints, which can limit their ability to prioritize and attend regular ANC visits. Additionally, with subsequent births, mothers may consider themselves more experienced and might underestimate the importance of early and frequent ANC visits (57). This self-satisfaction can lead to reduced utilization of maternal health services (58). *This assertion finds additional backing from research conducted in Ethiopia (44), and Cameroon (17). Although the study is cross-sectional, valuable policy insights can be drawn from the findings. Strengthening community-based interventions, such as targeted health education campaigns and home visits by community health workers, can improve awareness and encourage early ANC initiation. Integrating ANC services within existing maternal and child health programs and ensuring financial and geographic accessibility, particularly for socioeconomically disadvantaged groups, may reduce barriers to care. Recognizing women's right to access healthcare during pregnancy and childbirth is essential to reducing the risk of complications and ensuring equitable maternal health outcomes. Policymakers should consider revising national ANC guidelines to emphasize early initiation and continuity of care at the global standard, ensuring they are effectively disseminated and implemented across all healthcare facilities.*

The primary strength of our study was that we analyzed nationally representative data spanning over a decade, from 2007 to 2017–18. This extensive timeframe enabled us to explore the persistent factors contributing to late initiation and insufficient ANC visits among women in Bangladesh, which remain unresolved and urgently require attention. Notably, this is the first study of its kind to explore this relationship, utilizing multiple large, nationally representative datasets from Bangladesh Demographic and Health Survey (BDHS) pertaining to Bangladesh. Therefore, it paves the way for further studies in other LMICs in the future. However, several limitations should be noted. First, our study is observational and based on repeated surveys of different, yet representative, cohorts, which limits causal interpretation. Additionally, relying on self-reported data may introduce recall biases or social desirability biases. *Some participants may not accurately recall the date of first ANC visit or may over or underreport the number of ANC visits. This recall bias regarding the timing and frequency of ANC visits in self-reported data could affect the validity of the findings. Future studies with health linkage or health insurance data could validate self-reported data with medical records or conducting longitudinal data collection to prevent recall bias.* Employing a longitudinal design could better assess causal relationships and evaluate potential intervention strategies based on our findings. *Finally, future research can consider applying models such as Spatial Lag Models to address spatial autocorrelation of ANC services in Bangladesh.”*

4. What will be the author’s argument regarding women’s rights to access healthcare during

pregnancy and childbirth to reduce the risk of complications, as you mention that women with late ANC and low ANC have more complications during their pregnancy and the probability of MMR?

**Authors’ response**

Thank you for pointing this out. We have added the following paragraph in Discussion (page 19 of the revised manuscript) to address the point you raised,

“*Although the study is cross-sectional, valuable policy insights can be drawn from the findings. Strengthening community-based interventions, such as targeted health education campaigns and home visits by community health workers, can improve awareness and encourage early ANC initiation. Integrating ANC services within existing maternal and child health programs and ensuring financial and geographic accessibility, particularly for socioeconomically disadvantaged groups, may reduce barriers to care. Recognizing women's right to access healthcare during pregnancy and childbirth is essential to reducing the risk of complications and ensuring equitable maternal health outcomes. Policymakers should consider revising national ANC guidelines to emphasize early initiation and continuity of care at the global standard, ensuring they are effectively disseminated and implemented across all healthcare facilities.*”

5. Why can most women not access healthcare or ANC when women are more educated or the percentage of employed women are increased over the decade? Adding these arguments will enhance the discussion section. (Line 161- 163).

**Authors’ response**

Thank you for your suggestion. We have revised the discussion section, and it now includes the argument you mentioned (at the starting of 3^rd^ paragraph of discussion section),

*“Despite increases in women’s education and employment, many women in Bangladesh continue to face significant barriers in accessing healthcare and ANC. Issues including cultural norms, poor healthcare facilities, and financial challenges make it difficult for women in accessing healthcare facilities (40).”*

6. Suggest strengthening the discussion of risk factors for late and low ANC with additional

literature.

**Authors’ response**

Thank you for your suggestion. We have revised the discussion section for strengthening the discussion of risk factors for late and low ANC with additional literature form different LMICs,

*“Additionally, women facing challenges with distance to healthcare facilities showed higher odds of having a low number of ANC visits. Studies conducted in Burkina Faso (38), and Benin (39) further support to this finding.”*

*“Financial barriers significantly impact access to maternal healthcare services, as lower-income families may prioritize immediate basic needs over healthcare expenses, leading to delayed or irregular ANC visits(41,42). Evidence from studies in Ethiopia(43,44), and Cameroon (17) also supports this conclusion.”*

*“While most regions have shown improvement over the decade, regional disparities still exist, highlighting the ongoing need to address regional inequalities in maternal healthcare access in Bangladesh, as evident from recent studies(45). Regional variations in ANC utilization also evident in other LMICs like Ethiopia (46) and Indonesia (47).”*

*“Women with higher education levels had reduced odds of experiencing late initiation and insufficient ANC visits compared to those with lower levels of education which underscored the crucial role of mother’s education in promoting timely and adequate ANC attendance(41,48,49). This assertion finds additional backing from research conducted in other LMICs including Nigeria (50,51), Ethiopia (15,38,46), and Indonesia (47).”*

*“This self-satisfaction can lead to reduced utilization of maternal health services(58). This assertion finds additional backing from research conducted in Ethiopia (44), and Cameroon (17)”*

7. Why aren’t risk factors of maternal age discussed in the result and discussion section? (line 125 women’s age at birth).

**Authors’ response**

Thank you for your suggestion. We have revised both of the result and discussion section including maternal age. Please refer to the following text in page 20 of the revised manuscript,

“*A recent study in Bangladesh also highlights the crucial role of internet use, and mobile phone on reproductive health service (50). Maternal age also has been identified as a significant factor associated with both the timing and number of ANC visits. Previous study conducted in Ethiopia (51), Ghana (52), and Gambia (53) also found maternal age associated with timing and number of ANC visit.*”

8. Can you check the reference number 23?

**Authors’ response**

Thanks for pointing it out. We have revised this reference. After revising the entire manuscript, this manuscript is now listed as reference number 28 in the references section. The revised reference is,

*“Lumley T, Gao P, Schneider B. Package ‘survey’: Analysis of Complex Survey Samples. R package version 41. 2023;”*
